# Effect of Different Interventions to Help Primary Care Clinicians Avoid Unsafe Opioid Prescribing in Opioid-Naive Patients With Acute Noncancer Pain: A Cluster Randomized Clinical Trial

**DOI:** 10.1001/jamahealthforum.2022.2263

**Published:** 2022-07-29

**Authors:** Kevin L. Kraemer, Andrew D. Althouse, Melessa Salay, Adam J. Gordon, Eric Wright, David Anisman, Gerald Cochran, Gary Fischer, Walid F. Gellad, Megan Hamm, Melissa Kern, Ajay D. Wasan

**Affiliations:** 1 University of Pittsburgh School of Medicine, Pittsburgh, Pennsylvania; 2 University of Utah School of Medicine, Salt Lake City; 3 VA Salt Lake City Healthcare System, Salt Lake City, Utah; 4 Geisinger Health, Danville, Pennsylvania; 5 VA Pittsburgh Healthcare System, Pittsburgh, Pennsylvania

## Abstract

**Importance:**

Prescription opioids can treat acute pain in primary care but have potential for unsafe use and progression to prolonged opioid prescribing.

**Objective:**

To compare clinician-facing interventions to prevent unsafe opioid prescribing in opioid-naive primary care patients with acute noncancer pain.

**Design, Setting, and Participants:**

We conducted a multisite, cluster-randomized, 2 × 2 factorial, clinical trial in 3 health care systems that comprised 48 primary care practices and 525 participating clinicians from September 2018 through January 2021. Patient participants were opioid-naive outpatients, 18 years or older, who presented for a qualifying clinic visit with acute noncancer musculoskeletal pain or nonmigraine headache.

**Interventions:**

Practices randomized to: (1) control; (2) opioid justification; (3) monthly clinician comparison emails; or (4) opioid justification and clinician comparison. All groups received opioid prescribing guidelines via the electronic health record at the time of a new opioid prescription.

**Main Outcomes and Measures:**

Primary outcome measures were receipt of an initial opioid prescription at the qualifying clinic visit. Other outcomes were opioid prescribing for more than 3 months and a concurrent opioid/benzodiazepine prescription over 12-month follow-up.

**Results:**

Among 22 616 enrolled patient participants (9740 women [43.1%]; 64 American Indian/Alaska Native [0.3%]; 590 Asian [2.6%], 1120 Black/African American [5.0%], 1777 Hispanic [7.9%], 225 Native Hawaiian/Pacific Islander [1.0%], and 18 981 White [83.9%] individuals), the initial opioid prescribing rates at the qualifying clinic visit were 3.1% in the total sample, 4.2% in control, 3.6% in opioid justification, 2.6% in clinician comparison, and 1.9% in opioid justification and clinician comparison. Compared with control, the adjusted odds ratio (aOR) for a new opioid prescription was 0.74 (95% CI, 0.46-1.18; *P* = .20) for opioid justification and 0.60 (95% CI, 0.38-0.96; *P* = .03) for clinician comparison. Compared with control, clinician comparison was associated with decreased odds of opioid therapy of more than 3 months (aOR, 0.79; 95% CI, 0.69-0.91; *P* = .001) and concurrent opioid/benzodiazepine prescription (aOR, 0.85; 95% CI, 0.72-1.00; *P* = .04), whereas opioid justification did not have a significant effect.

**Conclusions and Relevance:**

In this cluster randomized clinical trial, comparison emails decreased the proportion of opioid-naive patients with acute noncancer pain who received an opioid prescription, progressed to treatment with long-term opioid therapy, or were exposed to concurrent opioid and benzodiazepine therapy. Health care systems can consider adding clinician-targeted nudges to other initiatives as an efficient, scalable approach to further decrease potentially unsafe opioid prescribing.

**Trial Registration:**

ClinicalTrials.gov Identifier: NCT03537573

## Key Points

**Question:**

Do clinician-targeted interventions prevent unsafe opioid prescribing in ambulatory patients with acute noncancer pain?

**Findings:**

In this randomized clinical trial of 22 616 opioid-naive adults with acute noncancer pain, providing primary care clinicians with email feedback on prescription practices resulted in a decrease in opioid prescriptions for acute noncancer pain compared with a guideline alone.

**Meaning:**

The results of this trial found that clinician comparison email feedback has the potential to decrease harm from opioid prescriptions for acute noncancer pain.

## Teaser

This cluster randomized clinical trial compares clinician-facing interventions to prevent unsafe opioid prescribing in opioid-naive primary care patients with acute noncancer pain.

## Introduction

Each year, up to 100 million individuals in the US experience clinically meaningful acute or chronic pain, mainly because of short-term illnesses, injury, and medical procedures.^1,2^ As such, it is imperative for health care clinicians and systems to offer patients effective treatment options that reduce symptoms and improve function. Nonopioid management is the preferred option^3,4,5^; however, there are circumstances (eg, severe pain, ineffectiveness of nonopioid medications) for which short-term opioid therapy is indicated and beneficial. The balance between these approaches is a problem in the management of acute noncancer pain.^6^

Most efforts to influence clinicians’ opioid prescribing, including the 2016 US Centers of Disease Control and Prevention and 2017 Veterans Affairs guidelines, have focused on treatment of chronic pain.^6,7,8^ Interventions are needed to improve the delivery of evidence-based, high-value care for acute pain. There is growing interest in using concepts from the fields of behavioral economics and psychology to nudge clinicians into providing evidence-based care.^9,10,11,12^ Furthermore, there is evidence for the use of behavioral science-based interventions to prevent inappropriate, guideline-discordant prescribing in similar clinical scenarios to the present study.^13,14,15,16^

We compared the effectiveness of clinician-targeted electronic health record (EHR) nudges and email feedback to encourage nonopioid management and prevent potentially prolonged and/or unsafe opioid prescribing in opioid-naive outpatients with acute noncancer pain. We hypothesized that, compared with a guideline alone, the addition of EHR nudges and email feedback would be associated with a decreased proportion of opioid prescriptions at the initial outpatient visit for acute noncancer pain and decreased progression to prolonged opioid prescribing or more than 3 months and concurrent use with benzodiazepines.

## Methods

### Trial Design

We chose a cluster-randomized clinical trial design to assess the interventions among a broad range of primary care clinical settings (Supplement 1 and Supplement 2). Cluster randomization was selected rather than simple randomization to minimize contamination and because the interventions being studied would likely be implemented on a practice-wide or system-wide level. The unit of randomization was the individual primary care clinic (cluster), and we used a 2 x 2 factorial design to test the interventions. The trial followed Consolidated Standards of Reporting Trials (CONSORT) reporting guidelines for randomized clinical trials.

### Trial Centers and Patient Inclusion Criteria

The trial was conducted in 48 primary care practices within 3 health care system participants in the PaTH Network, an affiliate of the National Patient-Centered Clinical Research Network (PCORnet). The 3 health care systems were University of Pittsburgh Medical Center (UPMC), Geisinger Health, and University of Utah Health. The 24 UPMC-affiliated primary care practices were in western Pennsylvania, the 13 Geisinger clinics were in central and northeastern Pennsylvania, and the 11 Utah practices were in the greater Salt Lake City area. The practices were a mix of internal medicine and family medicine. Patient enrollment began in September 2018 and continued through January 2020. Follow-up of patient data continued through January 2021. The University of Pittsburgh institutional review board served as the single institutional review board for the trial. A waiver of informed consent was granted for the trial as it involved no more than minimal risk, could not be conducted in real time without the waiver, and did not adversely affect the rights and welfare of the patient participants as the clinicians were free to deliver care as they believed appropriate.

We targeted an outpatient population with acute pain diagnoses (specifically*,* uncomplicated musculoskeletal pain and headache) for which opioids are rarely indicated but frequently prescribed.^3,17^ We used the following criteria to select participants: (1) inclusion criteria: older than 18 years and index outpatient encounter with *International Statistical Classification of Diseases and Related Health Problems, Tenth Revision (ICD-10)* acute neck, back, or other musculoskeletal and headache diagnosis (*acute* defined as no similar diagnosis during the past 6 months) (eTable in Supplement 3); and (2) exclusion criteria: cancer diagnosis (other than nonmelanoma skin cancer) and receipt of opioid prescription within 12 months of index outpatient encounter.

### Randomization

Practices were enrolled and randomized in a 2 x 2 factorial design to 1 of 4 comparator groups. The unit of randomization was the primary care clinic. Randomization was executed by providing the study statistician (A.D.A.) with a list of the primary care practices and intention to stratify practices by system and geographic status. The statistician prepared a random allocation that ensured approximately equal representation of practices and urban/rural practices in each of the possible treatment combinations.

### Interventions and Comparators

The trial used 4 groups: (1) control; (2) opioid justification; (3) clinician comparison; and (4) combined opioid justification and clinician comparison (groups hereafter abbreviated as control, justification, comparison, and justification/comparison). The interventions occurred during the flow of routine care, making it likely that patients did not notice any difference from routine care. Clinicians were not masked to the intervention arm that they participated in. For all groups, an alert to the clinician was triggered by clinician entry of a new opioid prescription for an opioid-naive patient (no opioid prescription during the previous 12 months) during an outpatient clinic encounter at a participating practice. In any of the comparator groups, the clinician was free to pursue the management strategy of their choice.

#### Control

When triggered by an opioid prescription during a qualifying visit, the alert contained a guideline with a short checklist of recommendations to (1) check the state’s prescription drug monitoring program; (2) assess risk factors for opioid-related harms (eg, history of substance use disorder, history of uncontrolled mental health problems, benzodiazepine use); (3) avoid extended-release or long-acting opioids; (4) use a low dose of immediate-release opioid for short period (3-7 days); and (5) consider nonopioid management, such as acetaminophen, nonsteroidal anti-inflammatory drugs, and physical therapy. The EHR order sets (ie, order sets that contained all necessary components to accomplish an order for a medication, test, or consultation) were linked to enable ordering of nonopioid therapy. This constituted an active control group, which we believed was necessary given the status of the opioid epidemic at the time the trial was developed. Further, an active control group helped to potentially normalize the group across the 3 health care systems, where different opioid prescribing initiatives might be active.

#### Justification

In addition to receiving the previously described EHR guideline, clinicians in the justification group were required to enter a free-text justification for their decision to prescribe an opioid analgesic for the acute pain condition. This comparator group was similar to the accountable justification strategy used by Meeker et al^14^ in their antibiotic study and was based on social psychology research that indicates that individuals will act in line with norms and guidelines because of reputational concerns.^18,19,20^ The clinician was informed that the justification provided would be visible in the EHR. For this study, informatics governance groups requested that we not directly indicate that no justification was given if the clinician did not enter a justification.

#### Comparison

Comparison can influence clinicians’ clinical practice.^21,22,23,24,25^ Clinicians in the comparison group received the previously described EHR guideline as well as monthly feedback via email regarding initial opioid prescriptions for acute pain, adherence to safe opioid-prescribing guidelines, and the proportion of patients who received opioids for acute pain who transitioned to treatment with long-term opioid therapy (>3 months) (eFigure in Supplement 3) compared with other clinicians. As seen in the eFigure in Supplement 3, the email text primarily described the meaning of the metrics in the figure, whereas the bar graphs depict the clinician’s performance, average of other clinicians’ performance, and performance of the lowest (best) 10% of clinicians. Feedback emails were not sent until May 2019, several months after intervention implementation, to ensure an individual clinician had adequate qualifying patient encounters.

For justification/comparison, clinicians received the EHR guideline and both interventions, as described previously.

### Data Collection

We used the PCORnet Common Data Model (CDM)^26^ to define standardized individual-level variables at the 3 health care systems, allowing the project to track enrolled patient participants for the EHR-tracked outcomes across all 3 health systems. In addition, the CDM enabled us to collect patient demographic characteristics, diagnoses, and medications. Patient participant race and ethnicity were obtained through the EHR. The EHR pharmacy order data were assessed to verify opioid-naive status at the qualifying acute care encounter. The PCORnet CDM is based on the US Food and Drug Administration Sentinel Initiative CDM (http://www.sentinelsystem.org) and has been informed by other distributed initiatives.

All clinician characteristics (eg, age, years in practice) were obtained from clinical site administrators. All data were stored on the secure University of Pittsburgh Health Services Research Data Center server.

### Outcomes

The primary outcome was an opioid prescription (yes/no) at the qualifying clinic visit, measured via EHR and excluding buprenorphine, methadone, or antitussives. Other outcomes were prolonged opioid prescribing of more than 3 months and concurrent opioid/benzodiazepine prescription over 12-month follow-up.

### Sample Size Calculation

We based the power analyses on 2 group comparisons (χ^2^ test for binary outcomes) for simplicity. Because we calculated power for 2 groups as if each group had the sample size of 1 comparator group in the 2 x 2 factorial design, the power estimates can be applied for the comparison of any possible pair of the 4 comparator groups. We adjusted the power calculation for an intraclass correlation (ICC) of 0.01, the median ICC estimated for more than 1000 variables from the studies in primary care research.^27^ The ICCs for specific outcomes were 0.016 for opioid prescribing at the index visit, 0.029 for long-term opioid therapy (>3 months), and 0.031 for concurrent opioid/benzodiazepine use. To be conservative, we assumed a 20% loss to follow-up for all outcomes. All powers were estimated at a significance level of *P* = .05. For the primary outcome of the proportion of participants receiving an initial opioid prescription, the sample size was estimated to achieve 81% power to detect a 2.5% absolute decrease in a comparator group of interest if the other group had 20% initial opioid prescription, assuming an ICC of 0.01.

### Statistical Analysis

All statistical analyses were intention to treat. Mixed-effects logistic regression models were used to test the treatment effect(s) of interest; each logistic regression model included fixed effects for practice assignment to the justification intervention, practice assignment to the comparison intervention, practice geography (urban/rural), and study month, as well as random effects for practice and health system. We report results for the main effects model, which included a yes/no indicator for each of the comparison and justification interventions, but not a separate indicator for receiving both interventions. Per protocol, we had planned analyses that included incorporation of the opioid prescribing trends at participating practices for the 12 months preintervention and to calculate opioid morphine milligram equivalents. However, the EHR data extraction to accomplish these analyses was either incomplete or inaccurate. Because of the inability to obtain accurate data before the intervention period and the lower-than-anticipated opioid-prescribing rate, we had to fit a simpler model that omitted interactions with the intervention period. In addition, because of the lower-than-expected incidence of opioid prescribing and the many clinicians that treated relatively few study-eligible participants, we could not include an additional random effect for clinicians; thus, we only included random effects for practice and health system.

For the secondary outcomes of unsafe opioid prescription and transition to treatment with long-term opioid therapy, we used a mixed-effects logistic regression to compare these outcomes at 12 months following the same structure as the primary outcome models. We used SAS, version 9.4 (SAS Institute), for all analyses.

## Results

### Participating Practices and Patients

**Figure 1.**
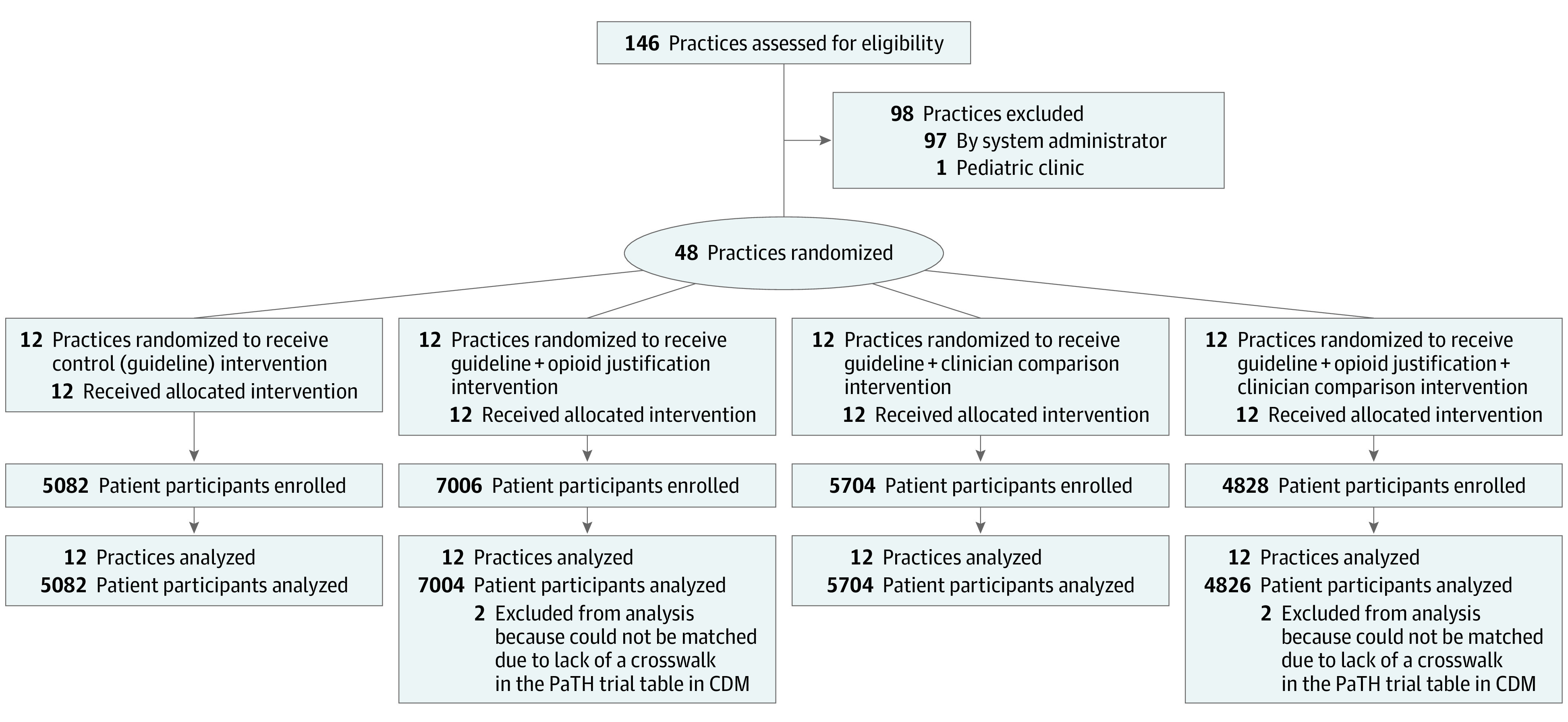
CONSORT Diagram of Primary Care Practice Participation CDM indicates the National Patient-Centered Clinical Research Network (PCORnet) Common Data Model.

Patients were enrolled from September 2018 through January 2020 (Figure 1). A total of 525 clinicians saw at least 1 qualifying patient during the intervention period and were included in the analysis. Of the 1 550 415 outpatient encounters at the participating primary care practices over the recruitment period, 22 620 patients (1.5%) met inclusion criteria. The median number of qualifying patients per practice was 375 (range, 92-1328). Of the 22 620 patients, 5083 (22.5%) were seen at practices assigned to control; 7004 (31.0%) at practices assigned to justification; 5705 (25.2%) at practices assigned to comparison (of these, 2926 [51.3%] were seen after the first comparison emails were sent); and 4826 (21.3%) at practices assigned to combined justification/comparison (of these, 2400 [49.7%] were seen after the first comparison emails were sent). Geisinger practices were somewhat underrepresented in justification and overrepresented in combined justification/comparison. Four patients were excluded from analysis because they could not be matched to CDM data, leaving a final sample of 22 616 for analysis. Enrolled patient participants had a mean (SD) age of 48.5 (17.7) years; 9740 (43%) were female, and 18 981 (84%) were White. Qualifying acute musculoskeletal pain conditions were similarly split among joint, spine, and soft-tissue conditions. Nonmigraine headache accounted for only 8% of qualifying conditions (Table 1).

**Table 1.** Sample Characteristics During Recruitment Period From September 2018 Through January 2020^a^

Characteristic	Overall	Control	Opioid justification	Clinician comparison	Opioid justification and clinician comparison
Practices, No.	48	12	12	12	12
Clinician characteristics					
Clinicians, No.	525	101	132	171	141
Qualifying patients/clinician, No. (range)	30 (1-265)	44 (1-206)	39 (1-211)	24 (1-265)	24 (1-214)
Age, mean (range)	46 (24-71)	45 (24-68)	47 (24-68)	46 (25-71)	46 (24-70)
Sex (n = 374)					
Female	195 (52.1)	35 (48.6)	44 (49.4)	52 (51.0)	64 (57.7)
Male	179 (47.9)	37 (51.4)	45 (50.6)	50 (49.0)	47 (42.3)
Years in practice, mean (range)	16 (1-58)	15 (1-45)	17 (1-58)	15 (1-43)	17 (1-42)
Clinic h/wk, mean (range)	33 (4-55)	35 (12-40)	34 (4-45)	31 (4-40)	33 (7-55)
Patient characteristics					
Patients, No.	22616	5082	7004	5704	4826
Age, mean (SD), y	48.5 (17.7)	48.2 (18.0)	48.8 (17.2)	47.3 (17.7)	49.9 (17.8)
Sex					
Female	9740 (43.1)	2278 (44.8)	2919 (41.7)	2409 (42.2)	2134 (44.2)
Male	12875 (56.9)	2804 (55.2)	4084 (58.3)	3295 (57.8)	2692 (55.8)
Not specified	1 (0.0)	0	1	0	0
Race^b^					
American Indian/Alaska Native	64 (0.3)	19 (0.4)	20 (0.3)	17 (0.3)	8 (0.2)
Asian	590 (2.6)	65 (1.3)	259 (3.7)	157 (2.8)	109 (2.3)
Black/African American	1120 (5.0)	391 (7.7)	329 (4.7)	276 (4.8)	124 (2.6)
Native Hawaiian/Pacific Islander	225 (1.0)	67 (1.3)	92 (1.3)	42 (0.7)	24 (0.5)
White	18981 (83.9)	4253 (83.7)	5568 (79.5)	4790 (84.0)	4370 (90.6)
Unknown	1636 (7.2)	287 (5.6)	736 (10.5)	422 (7.4)	191 (4.0)
Ethnicity					
Hispanic	1777 (7.9)	545 (10.7)	667 (9.5)	406 (7.1)	159 (3.3)
Non-Hispanic	19928 (88.1)	4379 (86.2)	5960 (85.1)	5117 (89.7)	4472 (92.7)
Unknown	911 (4.0)	158 (3.1)	377 (5.4)	181 (3.2)	195 (4.0)
Site					
University of Pittsburgh Medical Center	8469 (37.4)	1912 (37.6)	3134 (44.8)	1699 (29.8)	1724 (35.7)
Geisinger	5948 (26.3)	1422 (28.0)	930 (13.3)	1765 (30.9)	1831 (37.9)
Utah	8199 (36.3)	1748 (34.4)	2940 (42.0)	2240 (39.3)	1271 (26.3)
Qualifying diagnoses^c^					
Arthritis/joint pain (nonspine)	7602 (33.6)	1690 (33.3)	2340 (33.4)	1920 (33.7)	1652 (34.2)
Spine-related	6313 (27.9)	1330 (26.2)	2165 (30.9)	1552 (27.2)	1266 (26.2)
Soft tissue (eg, tenosynovitis)	7096 (31.4)	1578 (31.0)	2166 (30.9)	1784 (31.3)	1568 (32.5)
Musculoskeletal injury	1808 (8.0)	537 (10.6)	345 (4.9)	510 (8.9)	416 (8.6)
Nonmigraine headache	1847 (8.2)	422 (8.3)	577 (8.2)	449 (7.9)	399 (8.3)
Other	240 (1.1)	56 (1.1)	64 (0.9)	66 (1.2)	54 (1.1)

^a^ Data cells are No. (%) unless otherwise specified.

^b^ Race and ethnicity were extracted from the electronic health record.

^c^ Column percentages add up to more than 100% as some participants had more than 1 qualifying diagnosis.

### Primary Outcome: Opioid Prescribing at the Index Clinic Visit

**Figure 2.**
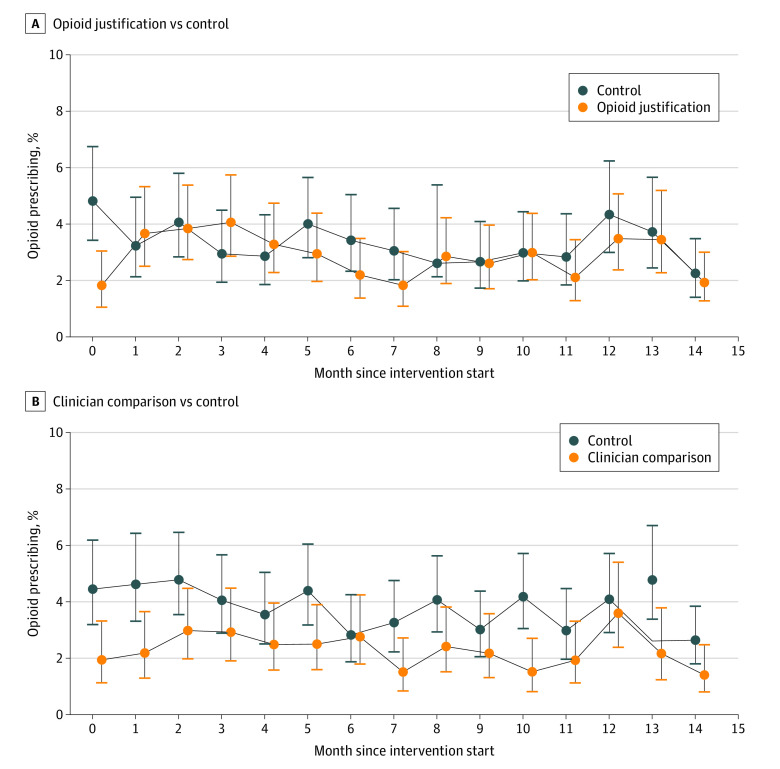
Rate of Opioid Prescribing at the Qualifying Clinic Visit A, Opioid justification vs control. B, Clinician comparison vs control. The figure shows opioid prescribing rates by month during the 15-month intervention phase of the trial. Month 0 is when interventions were initiated at the participating practices. The vertical lines indicate 95% CIs. Opioid justification includes participants from the justification and justification/comparison groups. Clinician comparison includes participants from the comparison and justification/comparison groups.

A total of 703 of the 22 616 qualifying patients (3.1%) were prescribed an opioid at the index study visit during the intervention period: 213 (4.2%) in control, 250 (3.6%) in justification, 150 (2.6%) in comparison, and 90 (1.9%) in justification/comparison. The opioid prescribing rates at the 3 health care systems at the qualifying clinic encounter was 1.0% at Geisinger practices, 2.9% at UPMC practices, and 4.9% at Utah practices and ranged from 0% to 9.5% at individual practices. Figure 2 illustrates opioid prescribing at the qualifying clinic visit for justification vs control (Figure 2A) and comparison vs control (Figure 2B). Across the 4 groups, when presented with the guideline after an opioid prescription was placed, clinicians proceeded with the opioid prescription 88.1% of the time (394 prescriptions of 447 instances), and only 13 (3%) opened the attached order set to order nonopioid management or medications.

The primary intervention comparisons are shown in Table 2. Opioid prescribing at the index visit was lower in the pooled comparison (main effects) model (adjusted odds ratio [aOR], 0.60; 95% CI, 0.38-0.96) throughout the total intervention period and after the comparison emails were sent.

**Table 2.** Intervention Effects on Opioid Prescriptions at Index Visit

Period	Opioid justification				Clinician comparison			
	Unadjusted^a^	*P* value	Adjusted^a^	*P* value	Unadjusted^a^	*P* value	Adjusted^a^	*P* value
Intervention period	0.79 (0.68-0.92)	.002	0.74 (0.46-1.18)	.20	0.57 (0.49-0.67)	<.001	0.60 (0.38-0.96)	.03
Before clinician comparison emails	0.82 (0.66-1.01)	.06	0.78 (0.45-1.35)	.37	0.59 (0.47-0.73)	<.001	0.60 (0.34-1.03)	.07
After clinician comparison emails	0.76 (0.61-0.94)	.01	0.70 (0.45-1.11)	.13	0.56 (0.44-0.70)	<.001	0.59 (0.38-0.93)	.02

^a^ Adjusted odds ratios estimated from mixed-effects logistic regression models that included fixed effects for practice assignment to the justification intervention, practice assignment to the comparison intervention, practice geography (urban/rural), and study month, as well as random effects for practice and health care system. Reference group: control.

### Prolonged Opioid Prescribing of More Than 3 Months and Concurrent Opioid/Benzodiazepine Therapy

Overall, 2424 (10.7%) of all participants and 527 (10.4%) of control, 902 (12.9%) of justification, 606 (10.6%) of comparison, and 389 (8.1%) of justification/comparison received an opioid prescription within the first 12 months after the qualifying visit. Additionally, 855 (3.8%) of all participants and 163 (3.2%) of control, 342 (4.9%) of justification, 223 (3.9%) of comparison, and 127 (2.6%) of justification/comparison progressed to treatment with long-term (>3 months) opioid therapy, whereas 651 (2.9%) of all participants, 112 (2.2%) of control, 263 (3.8%) of justification, 181 (3.2%) of comparison, and 95 (2.0%) of justification/comparison received opioids concurrent with a benzodiazepine prescription. Compared with control, comparison was associated with decreased risk of both outcomes in main effects models (Table 3).

**Table 3.** Intervention Effects on Long-term Opioid Therapy and Opioid/Benzodiazepine Concurrent Use During 12 Months of Follow-up

Period	Opioid justification				Clinician comparison			
	Unadjusted odds ratio (95% CI)^a^	*P* value	Adjusted odds ratio (95% CI)^a^	*P* value	Unadjusted odds ratio (95% CI)^a^	*P* value	Adjusted odds ratio (95% CI)^a^	*P* value
**Outcome: long-term opioid therapy (>3 mo)**								
Intervention period	1.13 (0.98-1.30)	.10	1.08 (0.94-1.24)	.26	0.85 (0.73-0.98)	.02	0.79 (0.69-0.91)	.001
Before clinician comparison emails	1.14 (0.95-1.26)	.17	1.12 (0.94-1.33)	.21	0.84 (0.70-1.01)	.06	0.82 (0.69-0.98)	.03
After clinician comparison emails	1.07 (0.85-1.35)	.58	1.01 (0.80-1.27)	.93	0.81 (0.63-1.02)	.08	0.73 (0.58-0.92)	.01
**Outcome: opioid/benzodiazepine concurrent use**								
Intervention period	1.15 (0.98-1.35)	.09	1.10 (0.94-1.29)	.25	0.96 (0.82-1.14)	.66	0.85 (0.72-1.00)	.04
Before clinician comparison emails	1.15 (0.93-1.42)	.19	1.13 (0.93-1.38)	.23	0.96 (0.78-1.18)	.67	0.88 (0.72-1.08)	.23
After clinician comparison emails	1.10 (0.84-1.43)	.49	1.03 (0.79-1.34)	.82	0.91 (0.69-1.20)	.51	0.78 (0.60-1.02)	.07

^a^ Adjusted odds ratios estimated from mixed-effects logistic regression models that included fixed effects for practice assignment to the justification intervention, practice assignment to the comparison intervention, practice geography (urban/rural), and study month as well as random effects for practice and health care system. Reference group: control.

## Discussion

In this cluster randomized clinical trial, compared with control, comparison was associated with reduced odds of an opioid prescription at the index visit, long-term opioid therapy (>3 months), and concurrent opioid/benzodiazepine therapy.

 Rates of opioid prescribing for this patient population were much lower than estimates derived during the trial protocol development. For example, opioid prescriptions at the index visit for this patient population from prior prescribing data were in the 15% to 20% range among practices in 1 of the participating health care systems. This observation mirrors national trends, with opioid prescriptions declining by 40% from their peak in 2011 to 2020,^28^ along with significant decreases observed in initial opioid prescriptions.^29^ Each of the participating health care systems used active opioid-prescribing initiatives during the study period, and clinicians were also exposed to state, federal, and payer initiatives, including monthly emails based on prescription drug monitoring program data and prior authorization for new opioid prescriptions. Such initiatives likely had an effect on clinicians’ practices, potentially overriding the effect of the interventions.

Despite the lower-than-anticipated rates of opioid prescribing and clinician exposure to multiple opioid prescribing interventions, we still observed statistically significant decreased odds of prescribing in the comparison groups, even before the initial comparison emails were sent. These results add to the growing literature that low-effect nudges can result in opioid prescribing changes and other health benefits,^24,25,30,31,32,33^ even in the context of likely concurrent interventions.

It is difficult to know the full deterrent effect of the nudges, as the simple EHR alert with guideline presentation and comparison emails may have prevented placement of an opioid prescription from the onset as clinicians were not masked to the interventions. Most clinicians (88%) continued with the opioid prescription even after the alert fired. We suspect that clinicians had made up their minds, having considered other options, before entering the prescription.

Although the rate of initial opioid prescription in the opioid-naive population was lower than anticipated, a significant number of patients received opioid prescriptions during 12 months of follow-up, progressed to treatment with long-term opioid therapy (>3 months), and had concurrent opioid and benzodiazepine prescriptions. These effects occurred in higher incidence in groups exposed to opioid justification, perhaps indicating a lack of duration of effect following initial prescription. These findings represent an opportunity to expand on this study and optimize nudge approaches to prevent unsafe opioid use and opioid misuse. Specifically, research could test several EHR strategies to alert outpatient clinicians to opioid risk and provide EHR tools that promote and simplify counseling of patients about opioid prescriptions, ordering of nonopioid treatment, education about and prescriptions of naloxone, and assessment for opioid use disorder criteria.^34^

### Strengths and Limitations

This trial had several limitations. First, the control group was an active control group. Practices in the control group received the guideline alert when an opioid prescription was entered. We believed that this was necessary given the burden of the opioid epidemic at the time the trial was developed. As a result, the counterfactual of no active intervention is unknown, perhaps reducing the magnitude of effect seen in other intervention arms. Second, practices were not masked to the comparator group to which their practice was assigned. This was offset partially by the active element of all 4 comparator groups and likely blanketed by the many EHR initiatives to which clinicians were exposed. The lack of masking may have contributed to the decreased odds of an initial opioid prescription in the comparison groups, even before the comparison emails were sent (ie, unmasked clinicians may have been anticipating the emails and adjusted their practice). It is possible that clinicians altered their coding practices to avoid best practice alerts, but we could not test for that possibility. Third, the recruited patient population was not racially and ethnically diverse, as 84% of patient participants were White, but this is consistent with the underlying population of the practice locations. Fourth, 1 of the health care systems (Geisinger) had a lower opioid prescribing rate (1%). Geisinger was overrepresented in the justification/comparison group but contributed similar numbers of patients to the 2 trial groups containing comparison overall. Although this potentially affected the findings, we statistically adjusted for practice and health care system in the models, which mitigated the imbalance. Finally, as discussed previously, limitations in data extraction prevented us from conducting planned analyses regarding 12-month preintervention opioid prescribing and calculation of morphine milligram equivalents for prescribed opioids.

This large trial also had some strengths. First, the trial delivered the interventions and measured outcomes in a manner least intrusive to primary care practice. Second, the trial recruited many patient participants in the 3 systems, including multiple urban, suburban, and rural practices. Lastly, the interventions are scalable and generalizable to systems of varying sizes.

## Conclusions

In this cluster randomized clinical trial, we found that clinician comparison emails decreased the proportion of opioid-naive patients with acute noncancer pain who received an initial opioid prescription. The rate of initial opioid prescription was several-fold lower than prior estimates, suggesting that local, state, federal, and payer initiatives were having a large effect on opioid prescribing practices. Health care systems can consider adding clinician-targeted nudges to other initiatives as an efficient, scalable approach to decrease potentially unsafe opioid prescribing.

## Notes

Supplement 1. Trial Protocol

Supplement 2. Statistical Analysis Plan

Supplement 3. **eTable.** Eligible ICD-10 codes for qualifying clinic visit **eFigure.** Example of Provider Comparison email

Supplement 4. Data Sharing Statement
