# Supplementary material for: Effect of Different Interventions to Help Primary Care Clinicians Avoid Unsafe Opioid Prescribing in Opioid-Naive Patients With Acute Noncancer Pain: A Cluster Randomized Clinical Trial
Source: JAMA Health Forum. 2022 Jul 29;3(7):e222263. doi: 10.1001/jamahealthforum.2022.2263 (PMC9338412; doi:10.1001/jamahealthforum.2022.2263)
Supplement: Supplement 1. — Trial Protocol [file jamahealthforum-e222263-s001.pdf]

Provider-Targeted Behavioral Interventions to Prevent Unsafe Opioid Prescribing for Acute Non-Cancer Pain in Primary Care

Study Protocol

Principal Investigator: Kevin L. Kraemer, MD, MSc, Professor of Medicine,
University of Pittsburgh

Funder: Patient Centered Outcomes Research Institute (PCORI)

Version 2
Date: October 29, 2021

Date of IRB Renewal Approval: April 14, 2021

ClinicalTrials.gov: NCT03537573

Table of Contents

PARTICIPATING STUDY SITES ... 6
STUDY TEAM ROSTER ... 6
University of Pittsburgh/UPMC ... 6
Geisinger Health System ... 7
University of Utah Health ... 7
ABSTRACT ... 8
1. STUDY OBJECTIVES AND SPECIFIC AIMS ... 9
2. BACKGROUND AND RATIONALE ... 9
3. STUDY DESIGN ... 13
4. SELECTION AND ENROLLMENT OF PARTICIPANTS ... 15
4.1 Practices ... 15
4.2 Providers ... 15
4.3 Patients ... 15
4.4 Study Enrollment Procedures ... 15
5. STUDY INTERVENTIONS ... 16
5.1 Guideline ... 17
5.2 Guideline + Opioid Justification ... 18
5.3 Guideline + Provider Comparison ... 18
5.4 Guideline + Opioid Justification and Provider Comparison ... 18
6. STUDY PROCEDURES ... 18
6.1 Randomization of Practices ... 18
6.2 Recruitment and Informed Consent Procedures ... 19
7. DATA COLLECTION AND QUALITY ASSURANCE ... 19
7.1 Electronic Health Record Data Collection ... 19
7.2 Follow-Up Surveys of 642 Patient Subset ... 20
7.3 Provider Demographics ... 20
7.4 Data Tracking/Reporting ... 21
7.5 Qualitative Interviews ... 21
7.6 Monitoring of Local Opioid Initiatives ... 23
7.7 Outcomes ... 23
7.8 Quality Assurance and Control ... 23
7.9 Data Security ... 24

8. STATISTICAL CONSIDERATIONS 24
8.1 General Approach 24
8.2 Analytic Plan for Specific Hypotheses 25
8.3 Power Calculations 27
8.4 Qualitative Analysis for Specific Aim 3 29
9. HUMAN SUBJECTS 30
10. ORGANIZATIONAL STRUCTURE 31
11. STAKEHOLDER ENGAGEMENT 32
12. DISSEMINATION 34
13. REFERENCES 36
14. SUPPLEMENTS/APPENDICES 40
Appendix A: Poster-Flyer in participating clinics 40
Appendix B: Information for Practices 41
Appendix C: Information Sheet for Providers 42
Appendix D: Clinic Letter to Patients (subset of 642) 43
Appendix E: Clinic Phone Call Script to Patients (subset of 642) 44
Appendix F: Phone Verbal Consent for Patients (subset of 642) 45
Appendix G: Online Web Consent for Patients (subset of 642) 47
Appendix H: Recruitment E-mail to Providers (random 100) 49
Appendix I: Phone Verbal Consent for Providers (random 100) 50
Appendix J: Online Web Consent for Providers (random 100) 52
Appendix K: EHR Data Extraction Computable Phenotype 54
Appendix L: Patient Subset (N=642) – Follow-Up Survey 55
Appendix M: Milestones/Timetable 56

Table of Figures

Figure 1. Study Design: pragmatic cluster randomized trial in 48 primary care clinics................ 13
Figure 2. Study Intervention Groups.. 17
Figure 3. Data flow between PaTH CDRN sites and Pittsburgh HSRDC 21
Figure 4. Outcome Comparison for Hypothesis 1a: Baseline (Qualifying Visit) 25
Figure 5. Outcome Comparison for Hypothesis 1b: Patient Reported Outcomes 26
Figure 6. Outcome Comparison for Hypothesis 2a: Patients Initiated on Opioids....................... 27
Figure 7. PCORI Pain Project Organizational Structure.. 31

Table of Tables

Table 1. Proposed project's compliance with PRagmatic Explanatory Continuum Indicator Summary-2 (PRECIS-2) guidance 14
Table 2. ERIC Strategies for Dissemination and Implementation... 34

PARTICIPATING STUDY SITES

University of Pittsburgh

University of Pittsburgh Medical Center (UPMC)/Community Medicine Incorporated

Geisinger Health System

Geisinger Community Practice Service Line

University of Utah

University of Utah Health Community Physicians Group

STUDY TEAM ROSTER

University of Pittsburgh/UPMC

Principal Investigator:
Kevin Kraemer, MD, MSc, Center for Research on Health Care, Division of General Internal Medicine, Department of Medicine, University of Pittsburgh School of Medicine, 230 McKee Place, Suite 600, Pittsburgh, PA 15213, Office: 412-692-4843, Fax: 412-692-4838, kraemerkl@upmc.edu

Project Manager:
Melessa Salay, MPH

Co-Investigators:

Brian Cohen, PhD (Patient Co-I)
Gary Fischer, MD
Jennifer Freel, MBA
Walid Gellad, MD, MPH
Megan Hamm, PhD
Andrew Althouse, PhD (Statistician)
Ajay Wasan, MD, MS

PaTH Personnel
Kathleen McTigue, MD, MPH (PaTH PI)
Barbara Postol (PaTH Project Manager)
Nickie Capella (Data Manager)
Bill Shirley (System Architect)
Waqas Amin (Informatician)
Hoah-Der Su (Source System Analyst)

Geisinger Health System

Site PI:
Eric Wright, PharmD, MPH

Clinical PI:
Christina Shook, PsyD, ABPP

Site Project Coordinator:
Melissa Kern, MPH

Site Research Assistant:
Cayla Pipan

Co-Investigators:
Michael Evans, RPh
Amy Howell-Harte, MD
Joseph Chronowski, MBA (Informatician)

PaTH Personnel:
Les Kirchner, PhD (PaTH PI)
Ilene Ladd, MS (PaTH Project Manager)
Dustin Hartzel (Data Manager)

University of Utah Health

Site PI:
Adam Gordon, MD, MPH

Clinical PI:
David Anisman, MD

Co-Investigator:
Jerry Cochran, PhD, MSW

Site Project Coordinator:
Connie Kitchen, PhD

PaTH Personnel:
Rachel Hess (PaTH PI)
Megan Lynch (PaTH Project Manager)
Reid Holbrook (System Architect)

ABSTRACT

Our project addresses the research question "What is the comparative effectiveness of different health system strategies that aim to prevent unsafe opioid prescribing while ensuring access to non-opioid methods for pain management with the goal of reducing pain and improving patient function and quality of life outcomes, while reducing patient harm?" We will assess whether behavioral science-based interventions can "nudge" providers towards more evidence-based care for patient with acute non-cancer pain.

Aim 1) Among opioid naïve primary care patients with acute non-cancer pain, compare the effect of the provider-targeted behavioral interventions (opioid justification and provider comparison), individually and in combination, on initial opioid prescription, initial use of non-opioid management, and patient-reported pain and function.

Aim 2) Among primary care patients who receive initial opioid therapy for acute non-cancer pain, compare the effect of the 2 provider-targeted behavioral interventions, individually and in combination, on unsafe opioid prescribing and transition to chronic opioid therapy.

Aim 3) Assess provider satisfaction and experience with the provider-targeted behavioral interventions.

Study Design: Pragmatic, cluster-randomized clinical trial in 48 primary care clinics.

Main Components: After implementing an evidence-based acute pain guideline in the electronic health record (EHR) at each of clinics, we will randomize the clinics to one of 4 intervention groups: 1) Usual Care; 2) Guideline + Opioid Justification; 3) Guideline + Provider Comparison; and 4) Guideline + Opioid Justification + Provider Comparison – all interventions delivered.

Study Population: The patient population will be 19,855 opioid naïve adults who present to clinic with acute uncomplicated musculoskeletal pain or headache.

Primary and Secondary Outcomes: The primary outcome measures will be receipt of an initial opioid prescription and unsafe opioid prescribing. Secondary outcomes will be non-opioid pain management, and, in 642 patients, patient-reported pain and function.

Analytic Plan: We will use statistical models to test for differences in the primary and secondary outcomes among the 4 intervention groups. We will use qualitative analysis methods to assess provider satisfaction and experience with the interventions.

Our multidisciplinary research team will work closely with a Stakeholder Advisory Committee comprising patients, patient advocates, primary care providers, pain medicine specialists, payers, health system executives, experts in behavioral science, and regional and national organizations. Once completed, the project may provide evidence that health systems and other stakeholders need to implement interventions to prevent unsafe opioid prescribing.

1. STUDY OBJECTIVES AND SPECIFIC AIMS

The overall objective of the research study is to compare the effectiveness of several provider-targeted electronic health record interventions to encourage non-opioid management and prevent unsafe opioid prescribing in outpatients with acute non-cancer pain.

With this pragmatic cluster-randomized trial, we will achieve the following specific aims:

Specific Aim 1) Among opioid naïve primary care patients with acute non-cancer pain, compare the effect of the provider-targeted behavioral interventions (opioid justification and provider comparison), individually and in combination, on initial opioid prescription, initial use of non-opioid management, and patient-reported pain and function at 1, 6, and 12months.

Hypotheses: Compared with usual care (guideline) alone, the addition of the opioid justification and provider comparison behavioral interventions will be associated with:

> Hypothesis 1a: Decreased proportion of opioid prescription and increased proportion of non-opioid management at the initial outpatient visit for acute non-cancer pain.
>
> Hypothesis 1b: No difference in patient-reported pain, function, and satisfaction at 1, 6, and 12 months.

Specific Aim 2) Among primary care patients who receive initial opioid therapy for acute non-cancer pain, compare the effect of the 2 provider-targeted behavioral interventions, individually and in combination, on unsafe opioid prescribing and transition to chronic opioid therapy (> 3 months).

Hypothesis 2: Compared with the usual care (guideline), the addition of opioid justification and provider comparison behavioral interventions will be associated with a decreased proportion of patients receiving unsafe opioid therapy and a decreased proportion of patients transitioning to chronic opioid therapy.

Specific Aim 3) Assess provider satisfaction and experience with the provider-targeted behavioral interventions.

2. BACKGROUND AND RATIONALE

The priority research question in the PCORI Funding Announcement (PFA) that our project addresses is "What is the comparative effectiveness of different payer or health system strategies that aim to prevent unsafe opioid prescribing while ensuring access to non-opioid methods for pain management with the goal of reducing pain and improving patient function and quality of life outcomes, while reducing patient harm?" This was the highest ranked research question (of 60 total) by a multi-stakeholder group at the "Preventing Opioid Misuse in the Management of Pain" PCORI workshop on March 7, 2016. Our focus will be on acute non-cancer pain as it presents in the primary care setting. Much prior research and many prior guidelines have focused on chronic non-cancer pain and chronic opioid therapy. There is a great need to develop effective approaches to acute non-cancer pain. Our local patient, provider, and health system stakeholders agreed this was an important question to focus

on for western Pennsylvania and the PaTH Future Research Topics Workgroup felt the same for the PaTH Network.

Acute non-cancer pain is very common. Up to 100 million individuals in the United States have pain annually, the majority due to short-term illnesses, injury, and medical procedures. Two of the most common acute non-cancer pain conditions, acute spinal (back and neck) pain and acute headache, are illustrative. Acute spinal pain accounts for over 10% of primary care visits in the US and approximately $86 billion in direct healthcare costs and $20 billion in indirect lost work productivity costs annually. Likewise, headache accounts for 12 million primary care visits per year and approximately $31 billion in direct healthcare costs annually. Because of the high prevalence and high societal costs of acute non-cancer pain, it is Because of the high prevalence and high societal costs of acute non-cancer pain, it is imperative for healthcare providers and systems to offer patients effective treatment options that reduce symptoms, improve function, facilitates return to activities, and prevents future problems due to over-prescribing. Depending on the specific cause of acute non-cancer pain and characterization of the pain as somatic, visceral, or neuropathic, treatment options may include non-opioid medications (e.g., acetaminophen, non-steroidal anti-inflammatory drugs (NSAIDS), gabapentin/pregabalin, serotonin and norepinephrine reuptake inhibitors (SSRIs/SNRIs), and tricyclic antidepressants (TCAs)), non-pharmacologic local measures (e.g., ice, heat, splinting, wraps, massage, tactile stimulation, acupuncture/acupressure), physical therapy, cognitive behavioral therapy, and opioid medications. All of these approaches, alone or in combination, can be effective in relieving acute pain. The evidence suggests that non-opioid management is the preferred option for the common pain conditions of headache, uncomplicated acute back, neck, and musculoskeletal pain, and self-limited illness (e.g. sore throat). For acute spinal pain and headache, recent national estimates indicate opioid prescribing rates of 29% and 18%, respectively. Although acute pain can often be managed without opioids, there are circumstances (e.g., severe pain, severe injury, post-surgical, ineffectiveness of non-opioid medications) where short-term opioid therapy is indicated and beneficial. Evidence suggests, however, that prescribing of opioid medications in the US has resulted in significant harm.

In the US, opioid prescriptions for non-cancer pain have increased several-fold in recent decades with no significant improvement in patient-reported pain and function. In 1991, 76 million opioid prescriptions were written in the US, rising steadily to 219 million opioid prescriptions in 2011, and falling slightly to 207 million in 2013, whereas other data estimate 259 million opioid prescriptions in 2012 and 245 million in 2014. Although the proportion of opioid prescriptions written for chronic vs. acute pain is not clear, 65% of opioid prescriptions in 2014 were for less than 3 weeks supply, suggesting the prescriptions were written for acute pain. Likewise, while it is not clear what proportion of patients who receive short-term opioid therapy transition to chronic opioid therapy, 9.6 to 11.5 million US adults received a prescription for long-term opioid therapy in 2014. The trend in increased use of opioids is associated with a current national public health crisis of opioid-related harms, including opioid misuse (using prescription opioids in any way other than as prescribed), opioid use disorders (defined by DSM-5), and non-fatal and fatal overdose. In 2015, 12.5 million misused prescription opioids, including 2.1 million new misusers that year, of whom 63% reported the opioid use was to relieve pain. Treatment for opioid use disorder increased along with the rise in opioid prescriptions from 199 to 2009. Between 2000 and 2014, the rates of prescription opioid overdose death nearly quadrupled from 1.5 to 5.9 deaths per 100,000 people. Although opioid prescriptions stabilized somewhat from 2010 to 2012, heroin use increased 36% from 2008 to 2013 and heroin, suggesting the possibility of former prescription opioid users switching to heroin. Among heroin users entering substance abuse treatment, 75% note their opioid use began with prescription opioids. Thus, the dramatic rise in opioid prescribing

for pain parallels the dramatic rise in opioid use disorders and fatal complications. The overreliance on opioids for acute pain fuels, in part, this public health crisis.

Although the response to the national opioid crisis must be multipronged and include healthcare, public health, community, law enforcement, and governmental resources, there is agreement that encouraging healthcare providers to adhere to safe evidence-based opioid prescribing practices should be a major part of the response. An effective strategy to help healthcare providers adopt non-opioid pain management strategies and, when opioids are deemed necessary, adhere to safe opioid prescribing practices for acute non-cancer pain has the potential to prevent downstream progression to chronic opioid therapy, reduce opioid related harms, and improve patient function and quality of life. However, despite this potential for improving short-term and long-term outcomes in patients with acute non-cancer pain, there are many gaps in evidence that must be addressed before interventions can be confidently implemented. Given the priority research question of our proposed project, we will focus on gaps in evidence for healthcare system approaches to opioid prescribing.

The majority of research and intervention development to date has focused on patients receiving chronic opioid therapy for chronic non-cancer pain. Some healthcare system interventions, such as those implemented by the VA and Kaiser Permanente, have resulted in decreased prescriptions for long-acting/extended-release opioids and reduced average morphine milligram equivalent dosing but it is not known if these reductions have resulted morphine milligram equivalent dosing but it is not known if these reductions have resulted in patient benefit. There has been minimal research and development of healthcare system interventions to encourage non-opioid management and, when needed, safer opioid prescribing in the acute stages of non-cancer pain treatment, when opioids are first prescribed or considered.

The optimal strategy for health systems to encourage providers to prescribe to non-opioid management and adhere to safe opioid prescribing guidelines for acute pain is not known. Provider-targeted interventions to decrease unsafe opioid prescribing have focused on chronic pain and have not been rigorously evaluated. For acute pain, just 1 of the 12 recommendations of the recent CDC Guideline for Prescribing Opioids for Chronic Pain focused on acute pain; CDC recommended that, if opioids are used, to use the lowest effective of an immediate-release opioid and to restrict prescription to short duration (recommended 3 days or less; more than 7 days rarely). This recommendation, while reasonable, was based on low quality evidence and health system interventions to encourage compliance with the recommendation have not been conducted. Some small, non-randomized studies of opioid guidelines for acute pain demonstrated decreased initial opioid prescriptions in emergency department and family medicine settings. Other guidelines, including the Institute for Clinical Systems Improvement (ICSI) "Acute Pain Assessment and Opioid Prescribing Protocol," have culled a low-quality evidence base to develop acute pain guidelines with good face validity but never tested in a rigorous fashion. As such, we do not know the optimal approaches to encourage providers to adhere to safe opioid prescribing and increase the use of non-opioid strategies while concurrently helping patients with pain and function.

Patient sub-groups and primary care clinic settings that benefit more and less from health system interventions for acute pain management is not known. There is little empiric evidence to suggest whether health system delivered behavioral interventions for acute pain management would be more effective in certain clinic settings or for certain patient populations. We speculate that such interventions may be less effective in rural clinics where non-opioid management strategies, such as physical therapy, might be less available and perhaps more effective among a higher risk (e.g., history of substance use disorder, mental health problems) patient population where the intervention encourages

the provider to be more cautious. However, there is little data on which to base a priori hypotheses. Instead, we plan exploratory analyses of a number of sub-groups. Provider and patient satisfaction and attitudes about health system provider-targeted behavioral interventions for acute pain management is not known. Although our intention is for the behavioral interventions to be low-burden, fit within the provider's work flow, and preserve autonomy, we do not know if providers or patients will perceive the interventions that way and be satisfied with their implementation. To address this gap, we plan qualitative interviews of a sample of providers and patients across the sites.

Significance

Providers do not always act rationally and deliver the most evidence-based care. Interventions to change provider behavior, including clinical guidelines, pay for performance, and computerized decision support have a modest history of success. In general, interventions to change provider behavior are challenging. EHR alerts and reminders can lead to "alert fatigue" and be ignored. Uptake of EHR-based guidelines and pathways also have low uptake and adherence. To improve the delivery of evidence based, high value care, there is growing interest in using concepts from the fields of behavioral economics and psychology to "nudge" providers toward providing evidence based care. Despite the scarcity of direct evidence to support the efficacy/effectiveness of behavioral nudges for opioid prescribing, there is compelling evidence for the use of behavioral science based interventions to prevent inappropriate, guideline-discordant prescribing in similar clinical scenarios to our proposed project. We have based our behavioral interventions on the work of several groups. Most influential to us was the recent cluster-randomized trial of Meeker et al. to reduce inappropriate antibiotic prescribing for upper respiratory infection among 14,753 outpatients. In that study, accountable justification and peer comparison reduced inappropriate antibiotic prescribing by 7% ($p < .001$) and 5% ($p < .001$), respectively, compared with control. The prescriber decision processes, guideline influence, and behavioral interventions are similar between the Meeker study and our proposed study. Given the similarity of the clinical decision, efficacy of the behavioral interventions, and urgency of addressing the opioid issue, we believe our planned comparators are appropriate for study in this context.

Our project and planned comparisons are highly important for patients, providers, and health system decision makers. We spoke with stakeholders from each group to confirm this. For providers, the health system intervention with the best patient outcomes and least workflow disruption and most preservation of autonomy will be the most ideal. The providers we spoke with recognized the role that early opioid prescribing could have on long-term opioid use but noted that opioid refills were too easy to write. Behavioral science based interventions have the potential to "nudge" providers toward guideline-concordant care and safer opioid prescribing while preserving autonomy and freedom of clinical decision-making. For health system decision makers, there is a definite decision dilemma among interventions as choice of an intervention must balance effectiveness for improving pain symptoms and function and reducing harm, maintain physician autonomy and satisfaction without disrupting workflow, and be feasible, sustainable, and scalable. To prevent overburdening providers and decreasing efficiency of care, health systems must be judicious in choosing which EHR interventions/innovations to implement. Behavioral "nudges," if simple and well-designed, can be speedy, in the work flow of a busy provider, and effective.

In the treatment of acute non-cancer pain, reduced pain symptoms and improved function is of highest importance. Therefore, for patients, the intervention that yields the most effective, evidence-based care, with reduced pain and short- and long-term harm, and improved function will be the most ideal. Further, patients do not like to feel their provider is being compelled to make decisions based on

external factors (e.g., pre-authorization, mandates). Behavioral nudges can encourage high-quality evidence-based care while preserving the provider's freedom of management. The potential of this project to increase evidence-based non-opioid management, decrease unsafe opioid prescribing, improve patient symptoms and function, and decrease long-term harm makes it highly patient centered.

3. STUDY DESIGN

The project is a pragmatic cluster-randomized trial. Forty-eight (48) primary care clinics (24 UPMC clinics, 13 Geisinger clinics, 11 Utah clinics) will be randomized in a 2x2 factorial design to one of 4 provider-targeted intervention groups (described in detail below): 1) Guideline; 2) Guideline + Opioid Justification; 3) Guideline + Provider Comparison; and 4) Guideline + Opioid Justification + Provider Comparison. The electronic health record (EHR) based interventions will be applied in the participating clinics in a quality improvement fashion.

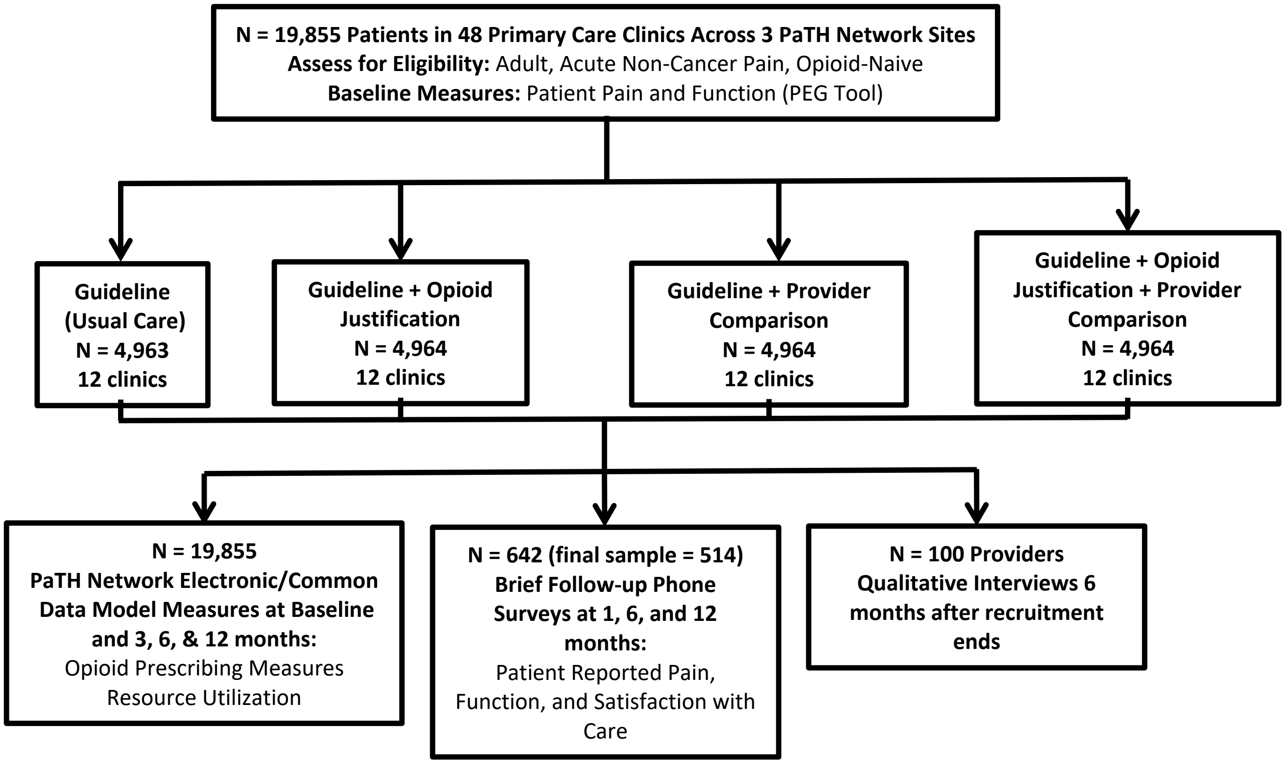

Figure 1. Study Design: pragmatic cluster randomized trial in 48 primary care clinics

Justification for Cluster Randomized Trial and 2x2 factorial design. We chose a pragmatic cluster-randomized trial as the most appropriate design because we wish to assess the behavioral interventions across a broad range of primary care clinical settings while minimizing contamination. The unit of randomization will be the individual primary care clinic (cluster). Stakeholders including the PaTH Network Future Research Workgroup Provider, health system administrators, and provider stakeholders agreed that a cluster randomized design was the most feasible design given the architecture of the Epic EHR (i.e., easier to enable a specific intervention feature for an entire practice site vs. for individual providers) and the need to avoid contamination among providers at a specific practice. A major advantage of 2x2 factorial design is the ability to test the usual care (guideline) versus opioid

justification, usual care (guideline) versus provider comparison, and opioid justification versus provider comparison, while the standard 2-arm trial can make only one of those three comparisons. Also, if assuming no interaction between interventions (as was observed in the Meeker antibiotic study), we can test usual care (guideline) versus opioid justification and versus provider comparison using half the sample size of 2 separate 2-arm studies.

Adherence to Pragmatic Trial Design Principles. We have taken systematic steps in the design of this cluster-randomized trial to adhere carefully to principles of pragmatic trial design, as specified by the **PR**agmatic **E**xplanatory **C**ontinuum **I**ndicator **S**ummary-2 (PRECIS-2) tool. As shown in the Table below, our proposed project is highly pragmatic.

Table 1. Proposed project's compliance with PRagmatic Explanatory Continuum Indicator Summary-2 (PRECIS-2) guidance

PRECIS-2 Domain	Criterion to be pragmatic	Answer for the Project	Pragmatic Rating
Eligibility	To what extent are participants similar to those who would receive this intervention if it was part of usual care?	Clinic and patient participants are identical to those who would receive the intervention in usual care	5
Recruitment	How much extra effort is made to recruit participants over and above what would be used in usual care setting to engage patients?	Patients will be outside of usual care setting but will not be seen face-to-face and assessments will be minimal	4
Setting	How different are the settings of the trial from the usual care settings?	Trial is closely integrated into usual care setting	5
Organization	How different are the resources, provider expertise, and the organization of care delivery in the intervention are of the trial from those available in usual care?	Trial is designed for interventions to be integral part of usual care	5
Flexibility (delivery)	How different is the flexibility in how the intervention is delivered and the flexibility anticipated in usual care?	Although interventions are targeted at provider behavior, the provider's decision-making and behavior is not constrained	5
Flexibility (adherence)	How different is the flexibility in how participants are monitored and encouraged to adhere to the intervention from the flexibility anticipated in usual care?	There is no difference in adherence monitoring from that anticipated in usual care	5
Follow-up	How different is the intensity of measurement and follow-up of participants in the trial from the typical follow-up in usual care?	We will ask a sample of 100providers to complete a qualitative interview at the end of the study and a sample of 642 patients to each complete 3 brief telephone surveys	4
Primary Outcome	To what extent is the trial's primary outcome directly relevant to participants?	For patients, the primary outcome (initial opioid prescription) is relevant: decreased opioid receipt may reduce downstream opioid risks but maintain pain relief and functioning	5
Primary analysis	To what extent are all data included in the analysis of the primary outcome?	All data will be included	5
*PRECIS-2 Pragmatic Scale: 1 = high explanatory/low pragmatic, 5 = low explanatory/high pragmatic			

4. SELECTION AND ENROLLMENT OF PARTICIPANTS

4.1 Practices

Inclusion Criteria: i) Internal Medicine or Family Medicine Practice in primary care network of the 3 participating systems; ii) Use of Epic electronic health record; iii) More than one primary care provider (see section 4.2)
Exclusion Criteria: None

4.2 Providers

Inclusion Criteria: Primary care provider (MD, DO, PA, NP) at participating practice
Exclusion Criteria: None

4.3 Patients

Inclusion Criteria: i) Age 18 years or older; ii) Index outpatient encounter with ICD-10 code for acute neck, back, or other musculoskeletal and headache diagnosis ("acute" defined as no similar diagnosis in past 6 months).
Exclusion Criteria: i) Cancer diagnosis (other than non-melanoma skin cancer); ii) Receipt of opioid prescription within 12 months of index outpatient encounter

4.4 Study Enrollment Procedures

4.4.1 Practices

We will recruit 24 UPMC practices, 13 Geisinger practices, and 11 University of Utah Health Care primary care practices. There are more potential sites overall across the 3 geographic locations to provide qualifying patients than required for the successful completion of the trial. The 24 UPMC clinics will be recruited from UPMC Community Medicine Incorporated (100+ clinics, 450,000 patients, average size 4500 patients/clinic). The 13 Geisinger clinics will be recruited from the Geisinger Community Practice Service Line (43 clinics, 300,000 patients, average size 6976 patients/clinic). The 11 Utah clinics will be recruited from the University of Utah Health Community Physicians Group (11 clinics, 150,000 patients, average size 12,500 patients/clinic (includes children)).

Screening Procedures for Practices: All primary care Internal Medicine and Family Medicine practices in the 3 health systems above with be eligible. Upon project initiation, the PI will work directly with the site PIs, site Clinical Champions, and primary care network directors to contact, via phone call, email, and/or direct interaction (face-to-face meetings and primary care network meetings) with primary care clinic directors and providers to introduce the project (information sheet for practices attached), provide information, and ask for participation. We anticipate a mix of urban and rural practices across the 3 systems. Randomization of participating practices will be stratified by system and urban/rural. Once randomized, providers at practices will receive a brief description of the arm their practice is randomized to.

4.4.2 Providers

A subset of 100 providers across the 48 clinics will be selected and consented for the Aim 3 12-month qualitative interview.

Screening Procedures for Providers: All providers who see patients at the participating practices will be eligible. Once the 12 months of patient recruitment is done, we will contact a random sample of 100 providers (stratified by health system and study arm) by e-mail (Appendix H). The e-mail will contain a link to consent (Appendix J), which when clicked by the provider will alert the research team to contact the provider and schedule an interview. If no response to the e-mail, we will contact the provider by phone (Appendix I) to set up a time to further describe the project and schedule the interview. If contact is unsuccessful after 3 attempts, we will randomly select another provider to replace them. If this process has a low yield, we will contact all of the remaining providers (that were not previously included in the random sample) at the participating practices by email.

4.4.3 Patient Participants

We anticipate at least 19,855 qualifying opioid-naïve adult patients with acute musculoskeletal pain or headache over the 12-month recruitment period.

Screening Procedures for the 19,855 patients: Once the interventions are implemented in the participating clinics, the PaTH Network Data Managers for each of the 3 health systems (UPMC, Geisinger, Utah) will start running weekly reports, based on PCORnet Common Data Model data extracted from the Epic EHR, of patients seen in participating clinics that meet inclusion/exclusion criteria.(Appendix K) Patients meeting the inclusion/exclusion criteria will be entered by secure computerized transfer into the de-identified analytic data set at the secure University of Pittsburgh Health Services Research Data Center.

Screening Procedures for the 642 patient subset to complete brief 1, 6, and 12 month surveys: We plan to recruit a 642 patient subset of the 19,855 total sample. This will be patients who complete the baseline PEG tool, administered as part of regular clinical care. The local PaTH Data Manager team at each health system will apply the screening criteria for this subset during the weekly Epic EHR data extraction for each participating clinic in their system. A weekly list of eligible patients will then be available to the project personnel. There will be a poster/flyer (Appendix A) placed in each participating clinic to alert patients of the project and the possibility of being contacted.

5. STUDY INTERVENTIONS

The EHR-based quality improvement interventions will be programmed into the Epic EHR for the participating clinics by the local health system EPIC staff. For all comparator groups, the evidence-based guideline will be triggered in the EHR by provider entry of a new opioid prescription in an opioid-naïve patient (no opioid script in prior 12 months) during an in-person outpatient clinic encounter at a participating clinic. In any of the 4 comparator groups, the provider has autonomy and is free to pursue the management strategy of their choice.

5.1 Guideline

The Guideline group will be the closest in content and structure to typical Epic clinical decision supports and best practice alerts. The guideline will follow the recent CDC guidelines and, when triggered by an opioid prescription during a qualifying visit, will be delivered real-time in a short checklist of recommendations to: 1) check the state-specific Prescription Drug Monitoring Program; 2) assess risk factors for opioid-related harms (e.g., history of substance use disorder, history of mental health problems, benzodiazepine use); 3) avoid extended-release or long-acting opioids; 4) use a low dose of immediate-release opioid for short period of time (3-7 days); and 5) consider non-opioid management such as acetaminophen, non-steroidal anti-inflammatory agents (NSAIDS), and physical therapy. Epic EHR order sets will be linked to enable easing ordering of non-opioid therapy. The order sets (Smart Sets) for non-opioid management hew toward "active choice," a type of behavioral intervention where a more evidence-based choice is made easier than the non-evidence based option. Although the "active choice" aspect of this may seem like a "strong" usual care component in this context, it should be noted that the provider has already entered an opioid prescription that serves as their default choice.

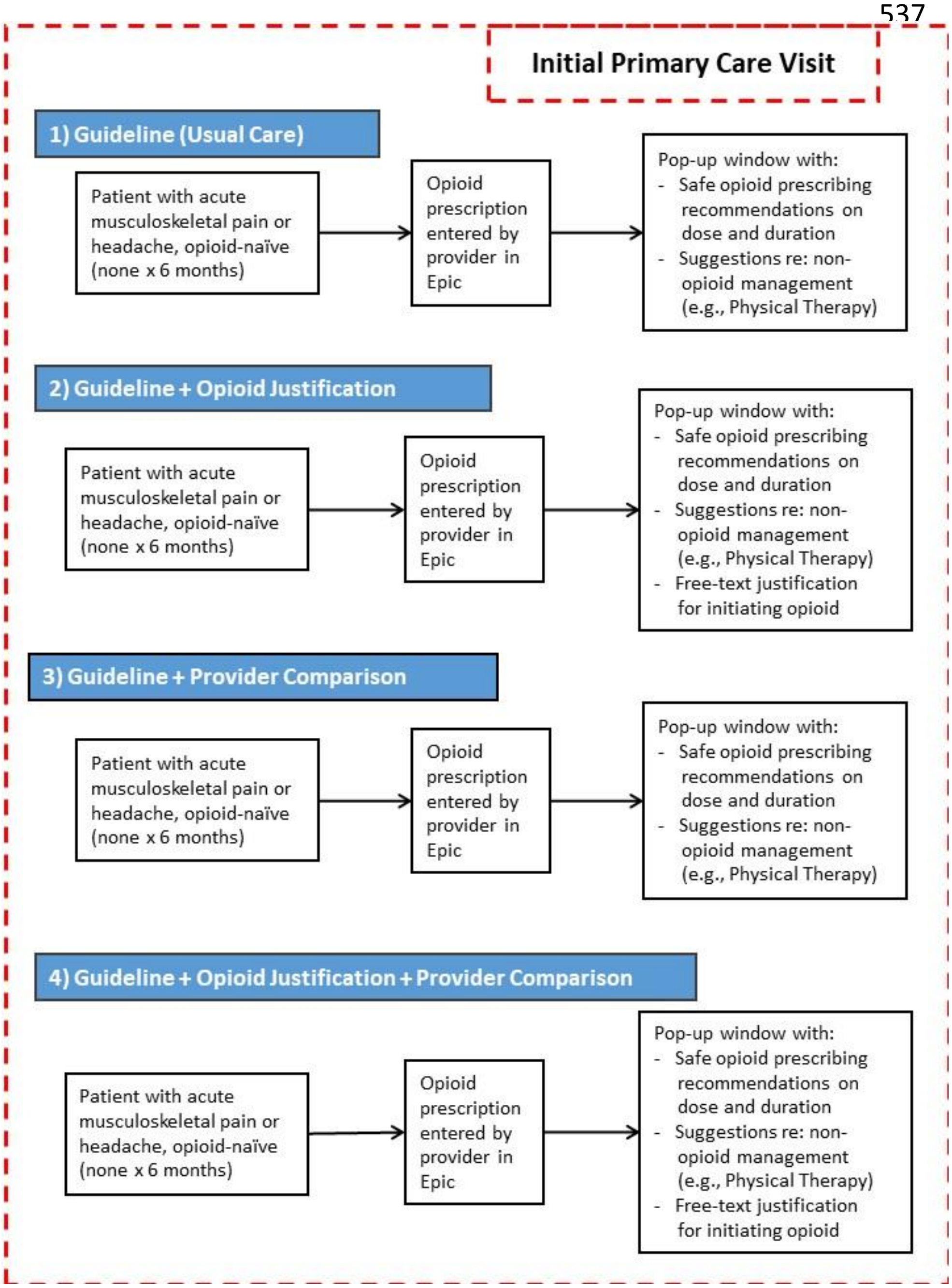

Figure 2. Study Intervention Groups

5.2 Guideline + Opioid Justification

In addition to receiving the Epic EHR guideline above, providers will be asked to enter a free text justification for their decision to prescribe an opioid analgesic for the acute pain condition. The provider will be notified that the justification provided will be visible in the Epic EHR. The provider has the option of entering a justification or not. If no justification is entered, nothing will be entered into the record (i.e., the Opioid Justification area in the encounter record will be left blank). The provider does not need to enter a justification if they choose to cancel the opioid prescription. This intervention arm is similar to the “accountable justification” strategy used by Meeker et al in the antibiotic study. This intervention is based on social psychology research that indicates individuals will act in line with norms and guidelines due to reputational concerns. That is, the strategy leverages the provider’s motivation to act within relevant norms, reflected by adherence to clinical guidelines, and desire to preserve their reputation, as reflected in the “public” opioid justification.

5.3 Guideline + Provider Comparison

In addition to receiving the Epic EHR guideline as described above, providers will receive monthly feedback via e-mail on their status in regards to initial opioid prescriptions for acute pain, adherence to safe opioid prescribing guidelines, and proportion of patients started on opioids website or acute pain who transition to chronic opioid therapy (> 3 months). Providers in the lowest decile overall for proportion of patients with initial opioid prescriptions , unsafe opioid prescribing, and transition to chronic opioid therapy (> 3 months) will be given positive feedback for providing high quality, evidence-based care to their patients with acute pain. Providers outside the “high quality” group will be notified they are outside the high quality, evidence-based care range and will be provided with their proportions compared to the average high performers’ proportions. Provider comparison feedback e-mails will not be sent for an individual provider until the provider has at least 20 qualifying acute pain patient encounters (see section 8). Again, the provider comparison intervention is similar to the Meeker et al. “peer comparison” arm. Although the provider comparison necessarily does not rise to the level of public “report card” disclosures, it goes beyond the usual feedback intervention in that it provides a high standard for the lower performers to strive for and gives enforcing feedback to the higher performers.

5.4 Guideline + Opioid Justification and Provider Comparison

Providers will receive the guideline and both behavioral interventions, as described in sections 5.1 to 5.3.

6. STUDY PROCEDURES

6.1 Randomization of Practices

We will randomize the 48 clinic sites to one of the 4 intervention arms. Given expected differences among the 3 PaTH geographic locations in local initiatives to address opioid prescribing, we plan to have even distribution of the interventions across the 3 systems. The unit of randomization is the primary care clinic. We will perform randomization stratified by health system and geography (urban vs. rural). Our rationale for stratified randomization is: 1) as discussed, each health system might implement new interventions/procedures to prevent unsafe opioid prescription, with potential impact on outcomes; and

2) prescription opioid misuse is higher in rural areas, possibly reflecting increasing propensity to opioid prescribing in rural areas. Dr. Althouse, the project's lead statistician, will conduct randomization using R software (R Foundation for Statistical Computing, Vienna, Austria).

6.2 Recruitment and Informed Consent Procedures

We will have a waiver to document informed consent for participants.

A) Patient Participants

Patients with qualifying clinic encounters will be tracked through the EHR by the local PaTH team. The interventions are minimal risk, targeted at providers, and occur in the flow of routine care, making it likely that patients will not notice any difference from routine care. We will request a waiver of informed consent for all 19,855 patients to undergo the research procedure of medical record abstraction. These outcomes will only be monitored via the the EHR and then transmitted in de-identified format to the University of Pittsburgh Health Services Research Data Center. The remaining 642 patients (randomly selected from those with baseline PEG data) will be sent a letter, signed by their local clinic director but sent by the local (Pitt/UPMC, Geisinger, or Pitt) study staff, after the qualifying clinic visit. In the letter, patients will be given the option to initiate phone or web consent procedures for the 1, 6, and 12 month brief telephone or web surveys or to actively decline to participate. We will request a waiver of signed informed consent for this 642 patient subset. A similar process is being used by the PCORI-supported multi-site TARGET low back pain trial, with IRB approval.

B) Provider Participants

We will randomly select 100 providers for the qualitative interviews. Randomization will be stratified by the 4 study arms and the 3 healthcare systems (UPMC, Geisinger, Utah). Stratification by study arm and healthcare system will systems (UPMC, Geisinger, Utah). Stratification by study arm and healthcare system will allow comparison of results across those factors. We will contact randomized providers by e-mail (Appendix H). The e-mail will contain a link to consent (Appendix J), which when clicked by the provider will alert the research team to contact the provider and schedule an interview. If no response to the e-mail, we will contact the provider by phone (Appendix I) to set up a time to further describe the project and schedule the interview. If contact is unsuccessful after 3 attempts, we will randomly select another provider to replace them. If this process has a low yield, we will contact all of the remaining providers (that were not previously included in the random sample) at the participating practices by email.

7. DATA COLLECTION AND QUALITY ASSURANCE

7.1 Electronic Health Record Data Collection

The majority of data will be collected through the PaTH network's established access to the Epic EHR of each of the 3 health systems. The PaTH Data Managers have the ability to query the Epic EHR and to extract health record data into datasets and to create reports. Through the PaTH/CDRN/PCORnet Common Data Model, we have defined, standardized individual level variables (see below) across the 3 PaTH sites, allowing the project to efficiently track the 19,855 patients for the EHR-tracked outcomes. It is important to note that data will be extracted for all 19,855 individuals who meet qualifying clinic visit criteria, not just the subset of patients who have an initial opioid prescribed. In addition, PaTH/CDRN

will easily enable collection of patient demographics, diagnoses, medications, outpatient and inpatient encounters, and services such as physical therapy. Pharmacy data will be assessed to verify opioid naïve status at the qualifying acute care encounter. For assessment of outcomes, only prescribing data will be collected.

The Common Data Model variables to be extracted include:

1) Baseline Visit Common Data Model variables for eligible patients: Age, Gender, Race, Ethnicity (Hispanic), Visit Diagnosis, Date, Tobacco, Diagnoses, Medications.
2) Among qualifying patients, proportion that receive a NEW (defined as no prior opioid prescription in prior 12 months) opioid prescription. Qualifying opioid prescriptions are oxycodone, hydrocodone, morphine, hydromorphone, oxymorphone, fentanyl patch, codeine, tramadol, methadone (including name, dose, #pills, follow-up fills for any opioid dispensed).
3) Baseline and follow-up variables: opioid medications (see above), non-opioid medications (e.g., NSAIDS, acetaminophen, tricyclic antidepressants, gabapentin, pregabalin, SSRI, SNRI, benzodiazepine, muscle relaxant), urine drug screens, physical therapy order, physical therapy visits, pain clinic referral order, pain clinic visits, behavioral therapy referral order, behavioral therapy visits, massage therapy order, massage therapy visits, follow-up outpatient visits and dx, emergency room visits and diagnosis, inpatient admission and diagnosis.

In addition, we will be able to track the Best Practice Advisory alerts that fire during the intervention.

The PaTH Network employs extensive security measures to ensure all patient information remains safe and private. The Common Data Model and study data sets are stored in the University of Pittsburgh's highly protected Health Services Research Data Center (HSRDC) (www.ccm.pitt.edu/health-services-research-data-center-hsrdc) and analyzed remotely via secure virtual desktops. The flow of data between the PaTH sites and the HSRDC is depicted in the flow chart below. The PaTH data pulls from the local sites will be done weekly for recruitment purposes and quarterly for data collection purposes during the project period.

7.2 Follow-Up Surveys of 642 Patient Subset

For a subset of 642 patients who complete the PEG at baseline, we plan to conduct very brief (< 5 minutes) surveys where the 3-item PEG and a single pain management satisfaction question (Appendix L) are administered at 1, 6, and 12 months after baseline. This will be done primarily via a secure custom web-based online survey system that will send reminders for completion before each survey. For individuals who do not wish to use the online system, we will offer the option of a brief phone survey with our research staff. It is necessary to offer phone survey in addition to the online survey because some patients will not be comfortable with electronic communication and we do not wish to bias the sample.

7.3 Provider Demographics

We will collect provider demographics (age, gender), specialty, years since first licensure, and clinic hours per week for providers at each participating primary care clinic and use a de-identified provider-specific code to link these data to specific patients.

7.4 Data Tracking/Reporting

The PaTH Network and Epic EHR teams at each site will pull and collate data regularly from the EHR and prepare reports. These include tracking of the total numbers of opioid-naïve patients for whom EHR best practice alerts fired, of subjects with qualifying outpatient visits, and total numbers of subjects with completed data elements, including the PEG instrument. Sites have agreed to conduct regular sweeps of the EHR with a report that documents total patients meeting eligibility criteria. These will be used to assure that we are recruiting at appropriate levels at all sites. The PaTH IT team at each site will do weekly pulls of data to track eligible patients and for recruitment of the 642 patient subset and quarterly pulls of the requested common data elements, de-identify them, and send to the prime site (Pittsburgh), where the HSRDC team and Data Integrity Committee will work to harmonize all of the data integral to the primary aims of the study from the 3 sites. Data from these resources will allow us to capture comprehensive information regarding opioid prescribing and other health care utilization for each patient in the study.

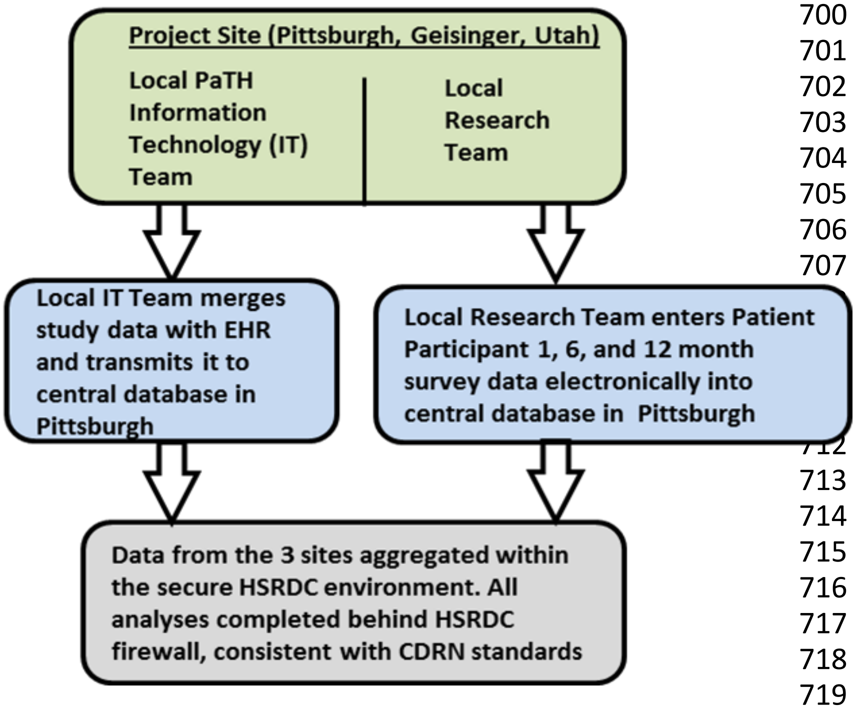

Figure 3. Data flow between PaTH CDRN sites and Pittsburgh HSRDC

7.5 Qualitative Interviews

Qualitative interviews, coding, and analyses will be conducted by the Qualitative, Evaluation and Stakeholder Engagement (Qual EASE) research service at the University of Pittsburgh Center for Research on Health Care Data Center. Qual EASE is directed by co-investigator Megan Hamm, PhD, a nationally recognized qualitative expert, and is staffed by master's-trained collaborators with three years' experience working for Qual EASE. Dr. Hamm has provided current Qual EASE staff with training in best interviewing practices (as per Dr. Hamm's experience, and as described in Michael Patton's Qualitative Research & Evaluation Methods). Additionally, when new projects are initiated, Dr. Hamm oversees piloting of new interview guides with Qual EASE staff, and listens to and provides feedback on the first 2-3 interviews that each staff member conducts, to ensure that the guide is being followed well and that interviewers are asking appropriate follow-up questions.

In order to better understand the viewpoints of the providers, we will conduct 100 half-hour long telephone interviews with 100 providers, starting 6 months after all patients have been recruited at their acute pain episode. This will prevent the qualitative interview from contaminating or influencing

provider decision-making during the acute pain care episode but still be fresh enough to assess implementation processes. We will identify willing providers from practices in all sites. Interviews will be open-ended and scripts will include perceived barriers to the intervention, satisfaction with care delivered, perceptions of success or failure and the degree to which providers believed they had to deviate from what they consider normally delivered care or perceived impact on autonomy. We will also explore provider thoughts about dissemination and implementation of findings and how they would like to receive them. The pilot qualitative interview script for providers will be developed in collaboration with co-investigators and our Stakeholder Advisory Board. Qual EASE's typical interview guide development process is to ask the study team for a list of topics and questions that they think are relevant, following which Dr. Hamm develops an interview guide that allows questions to flow through events in temporal order (if relevant), and from general questions to more specific ones. Guides begin with general, open-ended questions that allow the interviewee time to respond with as little prompting as possible, following which we ask follow-ups if something was unclear, or to prompt discussion of a facet of the topic that did not arise spontaneously in the interviewee's response. Qual EASE interviewers are empowered to ask relevant follow-ups not included in the guide if necessary, and to adjust wording if individual words are confusing or distracting to the individual interviewee. Finally, we use the readability tools embedded in Microsoft word to test that the script is at an appropriate vocabulary level for the study population, and test the script aloud amongst ourselves to ensure that questions are as comprehensible when spoken aloud as they are on the written page.

All provider interview scripts will be pilot tested with five providers. We will use a cognitive interview approach to assess the wording and content of the interview. During our pilot of this interview script, we will pay careful attention to wording or phrasing that is unclear or causes confusion or distraction to the interviewee. Pilot interviewees will also be asked if there are changes they would make to the guide, and why they feel those changes would be helpful. Transcripts of the pilot interviews will be provided to the study team for feedback, to ensure that the questions are eliciting the type of response intended to the study team (or that, in the event that unintended response types are occurring, the information is still of use to the study). A final interview guide will be crafted in response to this feedback, but as previously stated, Qual EASE interviewers will be able to make individual adjustments as necessary. The Qual EASE team will review the pilot results with Dr. Hamm and will refine the patient and provider interview scripts accordingly. All provider participants will complete informed consent at the beginning of the project. The informed consent document will include information about the potential to be randomly selected for the qualitative interview later in the project period. We will randomly select 100 selected for the qualitative interview later in the project period. We will randomly select 100 providers for the interview. Randomization will be stratified by the 4 study arms and the 3 healthcare systems (UPMC, Geisinger, Utah). Stratification by study arm and healthcare system will allow comparison of results across those factors. We will contact randomized providers by e-mail and, if no response, by phone to set up a time to further describe and schedule the interview. If contact is unsuccessful after 3 attempts, we will randomly select another provider to replace them. If this process has a low yield, we will contact all of the remaining providers (that were not previously included in the random sample) at the participating practices by email.

All interviews will be conducted by a member of Qual EASE who will have extensive training in interview data collection. All interviews will be recorded with a digital audio recorder and transcribed verbatim by trained members of Qual EASE for the final data analysis.

7.6 Monitoring of Local Opioid Initiatives

Monthly throughout the project, the Clinical Champion at each site will record new opioid prescribing and pain management initiatives that are implemented, their key features, and start and end dates.

7.7 Outcomes

The majority of the outcomes (listed below) are extracted from the EHR. Only the 3-item pain and function survey is from patient report.

Outcomes (Aim 1)

1) Initial opioid prescription (yes/no) (Primary) (Hypothesis 1a). Opioid prescription at qualifying clinic visit, measured via EHR.
2) Initial non-opioid management (yes/no) (Secondary) (Hypothesis 1a). Defined as order for any non-opioid management strategy at baseline (e.g., non-opioid medication, physical therapy, behavioral therapy), measured via EHR.
3) Patient Reported Pain and Function (Secondary) (Hypothesis 1b). Measured by the 3-item Pain/Enjoyment/General Activities (PEG) instrument in clinic at baseline and via brief telephone interview at 1, 6, and 12 months.

Outcomes (Aim 2)

1) Unsafe opioid prescribing (Primary) (Hypothesis 2). Unsafe opioid prescribing will be measured via EHR at 3, 6, and 12 months after the qualifying clinic visit. At each time point, it will be defined over the prior 3 months as any of: a) receipt of initial extended release/long-acting opioid for acute pain; b) > 100 morphine milligram equivalent dose per day; c) opioid prescription in patients with substance use disorder or concurrent benzodiazepine prescription.
2) Chronic opioid therapy (Secondary) (Hypothesis 2). Ongoing, chronic opioid therapy will be measured via EHR at 3, 6, and 12 months.

7.8 Quality Assurance and Control

The study will strictly adhere to safety and quality control procedures established by the Center for Research on Health Care Data Center at University of Pittsburgh. A data and safety monitoring plan will be implemented by the Principal Investigator to ensure that there are no changes in the risk/benefit ratio during the course of the study and that the confidentiality of research data is maintained. Investigators and study personnel will meet weekly to discuss the study (e.g., study goals and modifications of those goals; subject recruitment and retention; progress in data coding and analysis; documentation, identification of adverse events or research subject complaints; potential violations of confidentiality) and address any issues or concerns at that time. Minutes will be kept for these meetings and will be maintained in the study regulatory binder. Any instances of adverse events will be reported to the University's Institutional Review Board via established procedures

De-identified data may be made available to other researchers, PCORNet sites, PCORI, and other qualified local, state, federal, and private institutions to conduct scientifically justifiable analyses, for data sharing, and to monitor the project.

7.9 Data Security

Local identifiable data will be stored on secure password-protected servers at the research units of each of the 3 sites (University of Pittsburgh, Geisinger, University of Utah). De-identified coded data will be stored on the secure, firewall protected University of Pittsburgh Health Services Research Data Center and the University of Pittsburgh Center for Research on Health Care Data Center servers, consistent with PCORI Clinical Data Research Network, HIPAA, and FISMA standards.

Once recorded, the digital audio recordings of provider interviews will be stored on a secure, password-protected server at the University of Pittsburgh Center for Research on Health Care Data Center. No audio files will be kept on the digital audiorecorder. The digital audiorecorder will be stored in a locked file cabinet in a locked office suite within a locked building. Audio files will be identified by an individual study code and will not be linked with any personal identifiable data. The audio files will be destroyed once the qualitative analysis is done.

In the event of real or suspected electronic data interception, hack, or breach, we will immediately report the incident to the IRB/HRPO, University of Pittsburgh Computing Services and Systems Development Security, and to the funder (PCORI). We will take immediate steps to identify the nature, extent, and cause of the incident and take corrective measures. If the incident involved identifiable participant information, we will discuss with the IRB/HRPO and PCORI the best way to notify affected participants.

If any of the 642 patient subset and 100 providers who give verbal/web consent wish to withdraw from the study, we will give the individual the options of letting us maintain the data in the database for analysis or destroying the data. We will respect the individual's wishes. After the required data retention period, we will destroy the research records, identifiers, and linkage code information according to PaTH and Pitt Health Services Research Data Center policy.

8. STATISTICAL CONSIDERATIONS

8.1 General Approach

We will compare the distributions of baseline characteristics across the four intervention groups to assess the effectiveness of the randomization. All analyses for treatment group comparisons will use the original treatment assignment as randomized for each participant (intent-to-treat). We will adjust for the randomization stratification factors as well as baseline variables that either statistically or clinically differs across the four groups. Data transformations may be applied to outcomes depending on the shape of the distribution to better approximate normality. We will consider ease of interpretation and clinical meaningfulness when choosing transformations.
Our study uses a 2x2 factorial design, which evaluates the additive effects of two interventions compared to control in one experiment. For all outcomes, we do not anticipate an interaction between the two interventions will be present. That is, we believe the effect of *opioid justification* will be the same regardless of receipt of *provider justification* and vice versa. We based our assumption of no interaction between the interventions on the findings of a large, cluster-randomized trial of behavioral interventions to prevent inappropriate antibiotic use (Meeker, D., et al., *Effect of Behavioral Interventions on Inappropriate Antibiotic Prescribing Among Primary Care Practices: A Randomized Clinical Trial.* Jama, 2016. **315**(6): p. 562-70). The Meeker et al trial used a 2x2x2 factorial design to

randomize clinical sites to behavioral interventions (suggested alternatives, accountable justification, peer comparison) that were very similar to ours. They found no significant interactions between interventions. This ‘no interaction’ assumption on factorial design allows us to achieve the power to test the two interventions as if we run two separate trials (each with the same size) for each of the interventions. That is, we can have the power as high as the case where we recruit twice as many patients to run two separate trials. However, to rule out the possibility of interaction completely, we will test for interaction for each outcome. In the unlikely event of significant interaction, we will include the interaction term in the model to estimate the intervention effect precisely. The analysis plan below assumes that an interaction between *opioid justification* and *provider comparison* was not detected.

8.2 Analytic Plan for Specific Hypotheses

Aim 1, Hypothesis 1a: Compared with the guideline (usual care) alone, the addition of the opioid justification and provider comparison behavioral interventions will be associated with a decreased proportion of opioid prescription and increased proportion of non-opioid management at the initial outpatient visit for acute non-cancer pain.

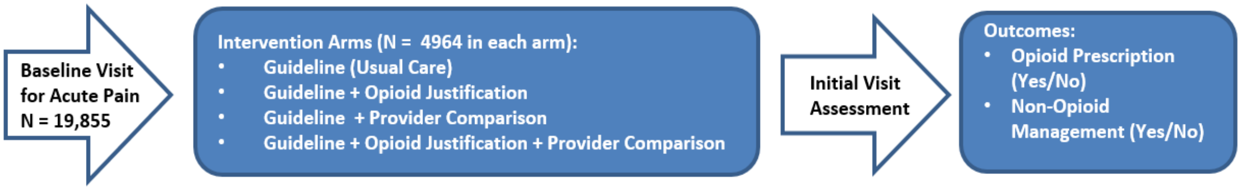

Figure 4. Outcome Comparison for Hypothesis 1a: Baseline (Qualifying Visit)

The primary outcome, initial opioid prescription, is expected to decrease in all four intervention groups and our goal is to detect the difference in change in the outcome across the four groups. For our primary analysis, we will use logistic regression to compare the initial opioid prescription after the intervention initiation across the four groups, with baseline opioid prescription rate at each clinic (during 1 year before the intervention) and other important clinical characteristics as covariates. To investigate further the effect of the interventions given the changing outcome rates in the background, we will use piecewise mixed effect logistic regression with a knot at month 0 (intervention start date). Fixed effects for this model will include an intervention group indicator (guideline (usual care) as reference group, a dummy variable for opioid justification, and another dummy variable for provider comparison), time (in month), intervention period indicator (1 after intervention starts, 0 before intervention starts) time since intervention start (in month), interaction between [opioid justification and (intervention period indicator)], interaction between [provider comparison and (intervention period indicator)], interaction between [opioid justification and (time since intervention start)], interaction between [provider comparison and (time since intervention start)], interaction between [opioid justification and provider comparison and (time since intervention start)], stratification factors, and other clinical covariates as fixed effects. We will include random effects for providers, clinics, and health systems to allow for clustering effect within each provider, clinic and health systems. If any of the four interaction terms (intervention period indicator × opioid justification, intervention period indicator × provider comparison, time since intervention start × opioid justification, time since intervention start × provider comparison) turns out to be significant, we can infer that the rate of initial opioid prescription either had sudden drop or decreased faster in the corresponding intervention group than the guideline (usual care) group.

To evaluate for changes in effectiveness over time, we will create a line graph for the primary outcome (opioid prescription rate) over time and visually inspect the data. If the EHR reminders become less effective, the decline of opioid prescription rate will become slower later than at the beginning of the study, which will be reflected in the line graph as the change of slope after a while since the intervention initiation. If the change in slope is suspected, we will identify the time point (=t1) that slope changes from the line graph, and include a term (time since t1 × intervention group) in the final model (piecewise mixed effect logistic regression model we proposed) to test it statistically. If the coefficient of this term turns out to be positive and statistically significant, we can say that after t1, the effect of EHR reminders decreased significantly.

Aim 1, Hypothesis 1b: Compared with usual care (guideline) alone, the addition of the opioid justification and provider comparison behavioral interventions will be associated with no difference in patient-reported pain, function, and satisfaction at 1, 6, and 12 months.

Patient reported pain and function will be compared using a linear mixed model with PEG as the outcome variable, and baseline PEG, intervention group indicator, time point, the interaction term between intervention group indicator and time point, stratification factors and other clinical factors as fixed effects. Random effects for patient, provider, clinic, and health system will be included as well to adjust for intraclass correlation within each patient, provider, clinic, and health system. Since our hypothesis is "*No difference in patient-reported pain, function, and satisfaction at 1, 6, and 12 months,*" we intend to perform equivalence tests on the coefficients of intervention group indicator and of the interaction term between intervention group indicator and time point. In order to do this, for each coefficient, we will perform two 1-sided tests using a slightly different t-statistics from that of standard t-test for regression coefficients, outlined as in Mascha and Sessler, 2011. If both tests are significant, we will conclude equivalence of the two groups that the corresponding coefficient represents the contrast of. Using the EHR, we will carefully assess for differences in demographics, clinic characteristics, healthcare system, and study arm between those with and without missing baseline PEG data and those with PEG data who do and do not consent to participate in the brief follow-up surveys. Understanding who is in the pain and functional outcomes portion (and who is not) will be important for reconciling the results of the reduction in opioid prescribing part of the project and the pain and function portion of the project.

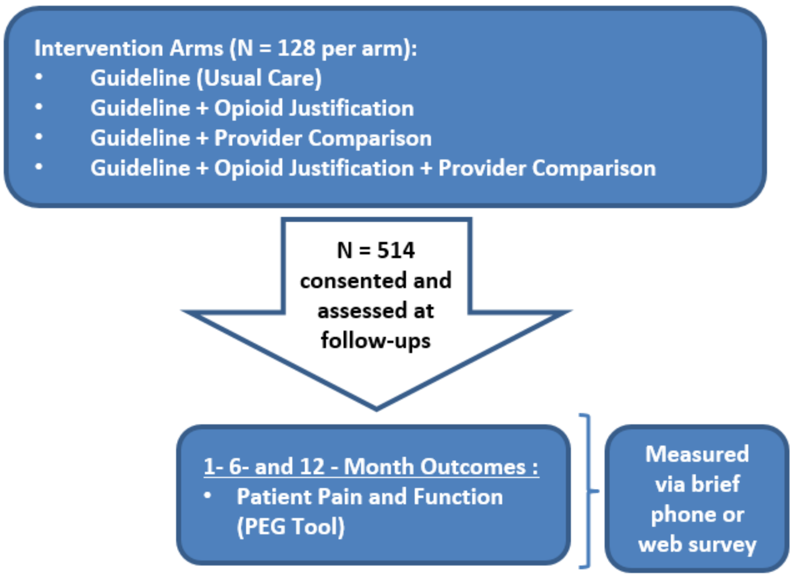

Figure 5. Outcome Comparison for Hypothesis 1b: Patient Reported Outcomes

Hypothesis 2: Compared with the usual care (guideline), the addition of opioid justification and provider comparison behavioral interventions will be associated with a decreased proportion of patients receiving unsafe opioid therapy and a decreased proportion of patients transitioning to chronic opioid therapy.

For hypothesis 2, we will assess outcomes in the total sample primarily and in secondary analyses restricted to those who were prescribed opioid at the initial visit (estimated 992 patients, 5% of baseline sample). (Figure 6 only shows plan for those prescribed opioid at initial visit). Unsafe opioid prescription and transition to chronic opioid therapy are binary outcomes which will be measured at 3, 6, and 12 months after each patient's initial visit. We will use mixed effect logistic regression to compare these outcomes at 3 months across the intervention groups. Fixed effects will include intervention group indicator, stratification factors and other important clinical factors. Providers, clinics and health systems will be included as random effects to adjust for the clustering effect within each provider, clinic and health systems. Outcomes measured at 6 months and 12 months will be analyzed in the same manner. For hypothesis 2 outcomes (unsafe opioid prescription and transition to chronic opioid therapy), the subgroup indicator and the interaction terms [intervention group indicator × subgroup indicator] for both *opioid justification* and *provider comparison* interventions will be included in the model. If these interaction terms turn out to be significant, it will indicate that the effect of intervention is significantly different between the subgroups.

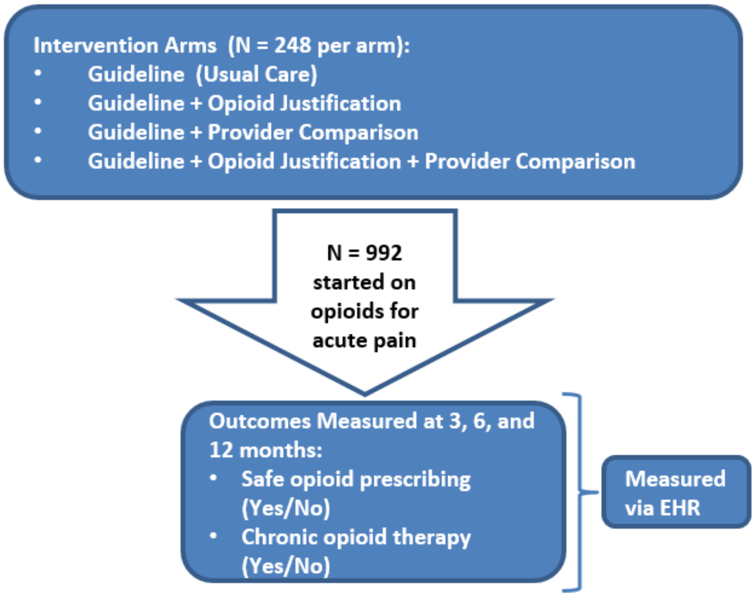

Figure 6. Outcome Comparison for Hypothesis 2a: Patients Initiated on Opioids

8.3 Power Calculations

Although the planned analyses are mixed effect linear or logistic regressions that compare the four intervention groups at the same time, we base our power analyses on two group comparisons (chi-square test for binary outcomes and equivalence t-test for PEG) for simplicity. Since we calculated power for two groups as if each group has the sample size of one intervention group in our 2x2 factorial design, the power estimates here can be applied for the comparison of any possible pair of the four intervention groups. However, if it turns out that there is no interaction between *opioid justification* and *provider comparison* as we expect, the effect of *opioid justification* will be evaluated using all of the 4 groups ([*guideline* group and *guideline* + *provider comparison* group] vs. [*opioid justification* group and *guideline* + *opioid justification* group]) rather than just *guideline* group vs. *guideline* + *opioid justification* group, so we will actually have greater power to detect the effect of *opioid justification* than presented below. Same argument applies to the effect of *provider comparison*. Since this is a cluster randomized trial and we do not have any preliminary data to inform us about the intraclass correlation coefficient (ICC) of our clinics, we adjusted the power calculation for ICC = 0.01 and 0.032, which are the median and 3rd quartile of the ICCs estimated for over 1000 variables from the studies in primary care research.[55] To be conservative, we assumed 20% loss-to-follow up (LTF) across all the outcomes except for PEG which will be based on n=642 participants who will be randomly selected from our cohort and followed up by email or phone call; with an expected final sample of 514 after an expected 20% attrition at 1 year (attrition rate based on experience of the similarly designed PCORI-supported

multi-site TARGET low back pain trial). All powers were estimated at significance level = 0.05. In regards to our assumption of equal cluster size, The 24 UPMC clinics will be recruited from UPMC Community Medicine Incorporated (100+ clinics, 450,000 patients, average size 4500 patients/clinic). The 13 Geisinger clinics will be recruited from the Geisinger Community Practice Service Line (43 clinics, 300,000 patients, average size 6976 patients/clinic). The 11 Utah clinics will be recruited from the University of Utah Health Community Physicians Group (12 clinics, 150,000 patients, average size 12,500 patients/clinic (includes children)). Except for the Utah clinics, we have not recruited the exact clinics that will participate but do have assurances from the leadership of the UPMC and Geisinger networks to recruit the final clinics, which will be a mix of urban and rural, large and small practices. As such, we do not have the exact mean number of patients and variation in hand. In the original application, we followed the recommendation of Campbell and Walters and used a coefficient of variation (standard deviation of cluster size divided by mean cluster size) in cluster sizes of 0.65 in our sample size calculations. [87]

In Aim 1, we expect to have total of n=19,855 patients in 48 clinics. Considering 20% LTF, this translates to 12 clinics in each intervention group with average of 331 subjects at each clinic. For % initial opioid prescription, our sample size will achieve 81% or 41% power to detect 2.5% absolute decrease in an intervention group of interest if the other group has 20% initial opioid prescription, assuming ICC = 0.01 or 0.032, respectively. For % initial use of non-opioid management, we can detect 10% increase in one intervention group with 99% or 75% power assuming the other group has 40% initial use of non-opioid management and ICC = 0.01 or 0.032. If 20% (n=642) of those with PEGs consent to complete the follow-up surveys, we will have 87%, 99.7% and > 99.9% power at baseline mean PEGs of 3.5, 5.0, and 6.5 to test for equivalence of PEG scores across intervention arms through 12 months after recruitment ends.

For the primary analysis power calculations, we used a simplified power calculation method of comparing two means or proportions between groups. This is commonly done in similarly designed studies (Meeker, D., et al., *Effect of Behavioral Interventions on Inappropriate Antibiotic Prescribing Among Primary Care Practices: A Randomized Clinical Trial.* JAMA, 2016. **315**(6): p. 562-70; Gerber, J.S., et al., *Effect of an outpatient antimicrobial stewardship intervention on broad-spectrum antibiotic prescribing by primary care pediatricians: a randomized trial.* JAMA, 2013. **309**(22): p. 2345-52). **For the more complicated task of power calculation when comparing slopes in the secondary analyses**, we simulated 1000 sets of data for each scenario, including potential interactions. We estimated power to detect the interaction between Opioid Justification and Provider Comparison by calculating [the number of simulated datasets that had β_5's p-value in M1 <0.05]/1000. Power to detect each main effect was estimated by [the number of simulated datasets that had β_{2A}'s (or β_{2B}'s) p-value in M2 <0.025]/1000. The results are summarized below. Indeed we do not have great power to detect the interaction. However, we have very good power (>99%) to detect the main effects, even when the clinically meaningful interaction effect exists.

Exploratory Analyses for Heterogeneity of Treatment Effects. As described earlier, we do not have *a priori* hypotheses regarding heterogeneity of treatment effects. There is little empiric evidence to suggest whether health system delivered behavioral interventions for acute pain management would be more effective in certain clinic settings or for certain patient populations. We speculate that such interventions may be less effective in rural clinics where non-opioid management strategies, such as physical therapy, might be less available, and perhaps more effective among a higher risk (e.g., history of substance use disorder, mental health problems) patient population where the intervention encourages the provider to be more cautious. However, there are little data on which to base *a priori* hypotheses. For this reason, we plan exploratory analyses in a number of *predefined sub-groups* of clinical and health

organization interest. Further, to detect an interaction effect the same size as our overall intervention effect would require a several-fold larger sample size, which would not be feasible within the budget constraints of the funding mechanism. For each of the following predefined subgroups (gender (male/female), age (< 65/> 65 years), opioid abuse risk (current or past diagnosis of substance use disorder and/or current mood disorder (anxiety/depression), Y/N), practice size (<4/≥4 providers), and practice location (urban/rural)), we will perform subgroup analysis using interaction terms in the model for the indicated contrasts. Using the urban/rural clinic variable as an example, we will first create the indicator variable for the subgroup of interest (urban/rural). For the outcomes in Aim 1 (initial opioid prescription and utilization of non-opioid pain management), each of these subgroup indicator variables and the interaction terms [time since intervention start × *opioid justification* × subgroup indicator] and [time since intervention start × *provider comparison* × subgroup indicator] will be included as fixed effects in the model. It certainly feasible to conduct similar *exploratory* analyses on the 2 clinical conditions, acute musculoskeletal pain and acute headache, and we will do so. We expect musculoskeletal pain to outnumber headache about 10,000 to 1,000. ***Missing Data*** *(MD-1-5).* We will compare baseline characteristics such as age, gender, and health insurance status between patients with complete follow-up data to those with missing data by treatment group in order to assess potential biases that may exist in the complete case analysis. We will conduct sensitivity analyses for the primary and secondary outcomes using several methods which have different missing data assumptions: (1) complete case analyses which assumes missing completely at random; (2) multiple imputation using M=10 imputations, which assumes missing at random; and (3) assigning poor scores and good scores for missing values differentially by treatment group, which aligns with non-ignorable missingness (the data missingness is related to the actual value).

8.4 Qualitative Analysis for Specific Aim 3

Qualitative analysis for provider interviews will follow the methods outlined by Crabtree and Miller. Crabtree and Miller describe several potential methods for analyzing qualitative data. We will draw on two of them: the "editing" process, followed by a "template" process. In "editing," qualitative analysts read through and engage with qualitative data, in this case interview transcripts, searching for meaningful segments of text that answer main research questions. "Editing" is an inductive process which allows the analysts to approach the data as much as possible without preconceived notions of what the results of the analysis will be. We will use this inductive process to create a "template," or codebook, to be applied to all of the interviews later in order to better organize the data. As such, Qual EASE staff, under the guidance of Dr. Megan Hamm, will begin reading transcripts in order to look for meaningful codes emerging from the data once half of the data has been collected. They will create codes that will be used to meaningfully categorize interviewee responses to questions. Because this process is inductive, it is not possible at this stage to give examples of codes that we will ultimately use. However, to give an example for clarification purposes, it is likely that we will create codes that describe barriers to using the proposed intervention (e.g., "Barriers to Use: Distraction Caused by Pop-Ups," "Barriers to Use: Pop-Up/Alarm Fatigue," "Facilitators to Use: Importance of Reducing Opiate Prescriptions," "Facilitators to Use: Pop-Ups Not Bothersome in Practice).

Codebook construction via the editing approach will begin after ½ of the qualitative data has been collected. A system of audit trails will be employed to document the creation of codes. A manual will be developed for each code in the new codebooks with specific inclusion / exclusion criteria for each code and textual examples of clear and borderline cases. Once Dr. Hamm and her team at Qual EASE feel that the codebook sufficiently describes the range of topics that arose in the interviews, that codebook will be applied to a randomly selected 20% of the interviews by two members of Qual EASE staff for the

purposes of corroboration of coding via Cohen's Kappa statistics. If the two coders have reached an average kappa score of 0.6 or above (regarded as "substantial agreement"), they will independently move on to code the remainder or the transcripts. If the two coders have not reached this level of corroboration, adjudication discussions led by Dr. Hamm will be conducted and additional transcripts will be coded to ensure consistent application of the codebook to all interviews. This process of coding independently (the basis for the intercoder reliability scores) and then discussing each case has enabled Qual EASE in previous research to maintain narrative coherence in the qualitative coding with an inter-coder reliability kappa scores of 0.75 and above. Once all interviews have been coded, a full quote report compiling all quotes relevant to individual codes will be produced. This quote report forms the basis of the thematic analysis of the interviews, allowing for consideration of the full range of meaning and experience behind each code, as well as for the consideration of relative frequency of codes. A full analytical report of patient and provider experience will be written by Dr. Hamm. Once the qualitative analyses are done, and in consultation with our Stakeholder Advisory Board recommendations, we will design and conduct mixed methods analyses where we compare outcomes (opioid initiation, non-opioid management, unsafe opioid prescribing, and progression to chronic opioid therapy) by qualitative domain.

9. HUMAN SUBJECTS

Institutional Review Board (IRB) Review. The University of Pittsburgh IRB will be the IRB of record for the project. Via the SmartIRB structure, the Geisinger and Utah IRBs will cede regulatory oversight to the University of Pittsburgh IRB. For this project: i) a waiver of consent and HIPAA will be used to collect medical record data on the 19,855 patients and to calculate background opioid prescribing rates; and ii) a waiver to document written consent will be used to enroll the 100 provider participants.

Risk. This a minimal risk study. The interventions are provider-targeted electronic health record (EHR)-delivered, evidence-based opioid prescribing guidelines or behavioral "nudges" (entering a justification for an opioid prescription and/or receiving periodic e-mail performance comparison on opioid prescribing practices). Research procedures are no more than minimal risk: i) the brief PEG questionnaire is commonly used in clinical settings and will likely take less than 1 minute to complete; it poses little risk to the patient; and ii) the qualitative interviews of providers are intended to obtain providers' thoughts about and experiences with the project's provider-targeted interventions. It will be a phone interview that poses little risk to the provider. Patient participants are unlikely to note any difference from a routine outpatient visit and the subset of patient participants who complete follow-up surveys will only have the inconvenience of completing a brief phone or web survey. Providers will notice additional EHR functions/windows delivered in the course of clinical care for certain patients and/or receive a periodic e-mail. Providers will have full autonomy to manage the patient as they deem appropriate. Because de-identified data will be tracked and transmitted to the Pittsburgh Health Services Research Data Center (HSRDC) and Pittsburgh Center for Research on Health Care Data Center, there is a small risk of loss of confidentiality.

Benefits. Patients may benefit from more exposure to evidence-based care and less exposure to potential harm from opioids. Providers may benefit from providing more evidence based care and improved outcomes in their patients. If the interventions are effective, there will be a public health benefit from decreased unsafe opioid prescribing.

Subject Safety. The safety of subjects enrolled in the study and the protection of privacy will be the responsibility of the investigators. All research interviewers will be trained in the recognition of psychiatric emergencies (e.g., acute homicidality, suicidality, opioid withdrawal symptoms and signs, or aggressive behavior) should unforeseen problems arise during the research assessments. All urgent problems or emergencies will be brought to the immediate attention of the Principal Investigator and Project Manager.

Staff Training. Each member of the study team will meet with the PI and review confidentiality issues and complete a confidentiality agreement, prior to having contact with research subjects. All investigators and research personnel have completed all required Research Practice Fundamentals modules. All research data with identifying information will be stored in locked files. Data will be recorded and identified by subject code numbers only in a properly secured computer database. Only members of the investigative group will have access to secured files. Identities of participants will not be revealed in publications or presentations derived from this project. Routine weekly and as-needed meetings between the PI and data staff will insure that these procedures are followed and that quality assurance measures are observed to insure data integrity and confidentiality.

Participant Compensation. For the subset of 642 patients for brief follow-up surveys, participants will receive $15 for each completed survey (maximum of $45 total if all 3 time points are completed). The 48 primary care practices will be reimbursed $1200 per provider, unless the practice expressly disallows such payments. The 100 providers who complete the qualitative phone interview will each receive a one-time payment of $50.

10. ORGANIZATIONAL STRUCTURE

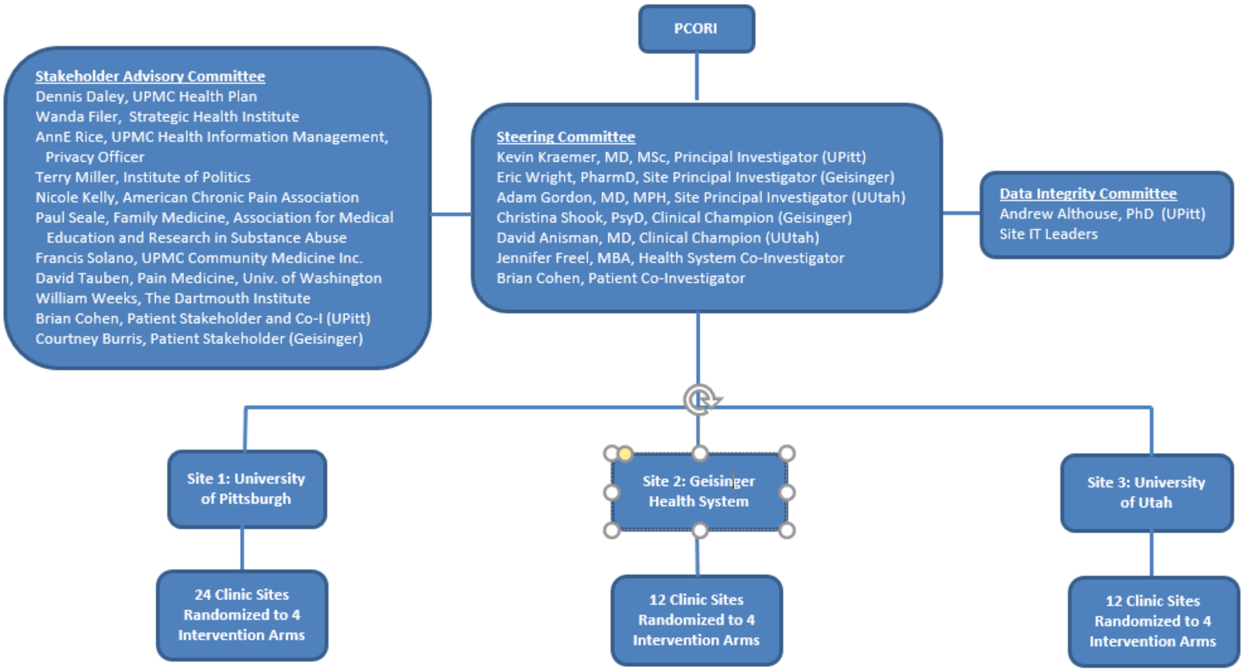

Figure 7. PCORI Pain Project Organizational Structure

11. STAKEHOLDER ENGAGEMENT

Planning the Study. This proposal was designed with input from multiple stakeholders, including patients with a history of acute pain. Early on, we valued the report from PCORI's multi-stakeholder "Preventing Opioid Misuse in the Management of Pain" PCORI workshop on March 7, 2016. The primary research question for our proposal was the highest ranked research question from that workshop. In preparing our LOI, we consulted with local patients and primary care clinicians, the PaTH Future Research Workgroup, clinicians at the PaTH sites, UPMC administrators and health information technology experts, and UPMC Health Plan senior administrators regarding the study design and interventions. Once the LOI was approved, we engaged national and local stakeholders, including patients, providers, payers, professional organizations, purchasers, and policy representatives to develop the full proposal. From those discussions, our Stakeholder Advisory Committee (Table next page) was formed. If funded, the Committee will work closely with the Steering Committee to develop and approve the Final Study Protocol prior to initiating the study.

Role of the Stakeholder Advisory Board. The role and function of the stakeholder advisory board is to: 1) help formulate and refine the research questions, study design, and procedures; 2) participate in and monitor the conduct of the project, including comparison of progress to the stated goals and deliverables in the Project Plan and Timeline; 3) help plan the dissemination of the project's findings and identify opportunities for outreach; and 4) make recommendations and troubleshoot problems. Our patient and stakeholder engagement plan is guided by five key principles as outlined in the PCORI Engagement Rubric: 1) building mutual trust by engaging patients and caregivers; 2) ensuring transparency by making all aspects of the research process open and understandable; 3) engaging in collaborative learning; 4) respecting patients and other stakeholders; and 5) fostering partnerships. Although the current Stakeholder Advisory Committee is primarily professionals, we anticipate adding patients and other stakeholder that will let us fully adhere to the principles above. We will strive for shared governance and follow a shared decision-making process in which all stakeholders are considered equal partners and have voice.

We will address the five key principles through the following ways:

i) We will build *trust* by engaging patients and other stakeholders, recognizing individual competencies and perspectives, encouraging and answering questions, and clarifying anticipated outcomes. We will suggest and obtain consensus among all research partners on ground rules for all meetings. These will emphasize active listening, not interrupting others, a spirit of mutual respect, and a designated opportunity for all meeting participants to weigh in with their opinions. This ensures an atmosphere of inclusivity and sensitivity to multiple perspectives.

ii) We will ensure *transparency* by making all aspects of the research process, including study design, data collection and analysis as open, understandable, and replicable as possible. In addition to the quarterly stakeholder meetings, we will maintain transparent communication for the duration of the project with a project website. The website will be disseminated to the entire stakeholder community and each member of the community will be invited to provide input on the site, which will be regularly updated with study-related news. The website will allow us to gain a much broader input from patients and PCPs throughout the duration of the project.

iii) We will ensure *collaborative learning* by engaging team members and stakeholders by sharing experiences, capitalizing on each other's resources, knowledge, and skills, evaluating each

other's ideas, and monitoring. This will be a continuous process woven into investigative team and stakeholder advisory committee meetings. Patient co-investigators will be asked to take a two hour online or live human subject protection training to enable them to be formally listed on the IRB application and participate in the research as needed.

iv) We will ensure *respect* for patients and stakeholders by acknowledging contributions and protecting confidentiality. Individuals will be encouraged to bring concerns to the PI and mitigation strategies will be developed on a case-by-case basis.

v) We will ensure *partnership* by creating an environment in which investigators, staff, and stakeholders all collaborate to advance their mutual interests from the earliest stages of the project through to dissemination of findings. Investigators, staff, and stakeholders will all be fairly compensated for their time, contributions, and travel.

Contact and communication between the research team and stakeholder advisory committee will be maintained between stakeholder meetings via e-mailed updates, as-needed phone calls if issues arise, and the project website. Advisory board members will have training in what is known and what is not known about the clinical topic of the project, behavioral "nudge" interventions, and how we will incorporate the 5 principles of engagement into the collaboration. We will have initial team learning sessions about patient-centeredness and stakeholder engagement. In-person meetings will be organized to ensure an inclusive, respectful, and transparent environment. A draft meeting agenda will be distributed well in advance of the meeting and all attendees will have the opportunity to comment or suggest changes to the content and structure. We anticipate the meetings will be a mix of large group and small breakout group activities to encourage participation. Leadership of groups will be rotated so all individuals will have a chance to take an active role. All discrete sessions will allow time for open discussion and reflection. To resolve disagreements, we will use standard conflict management strategies including: 1) open communication; 2) focus on behavior and issues, not personalities; 3) listening carefully; 4) identifying points of agreement and disagreement; 5) prioritizing issues; 6) developing a plan to resolve specific issue; 7) implement and follow through on the plan; 8) building on success; and 9) staying calm.

Completing the Stakeholder Advisory Committee: The Stakeholder Advisory Committee with work closely with the Steering Committee on all aspects of conducting and monitoring the study. The Stakeholder Advisory Committee and Steering Committee will have a face-to-face day-long meeting in Pittsburgh twice yearly and a telephone/videoconference meeting twice yearly for each year of the 3-year project. The Data Safety and Monitory Board (DSMB) will join the face-to-face meetings to monitor study progress, help troubleshoot problems, and compare study progress to the stated milestones and deliverables.

Disseminating the Results. We will involve the Stakeholder Advisory Committee in all aspects of dissemination and implementation. Activities will include: (1) identifying partner organizations for dissemination to ensure meaningful and direct communication with end-users; (2) planning dissemination efforts from the very beginning to be focused on the end product; (3) participating in dissemination efforts, such as co-authoring manuscripts and delivering oral presentations, to offer the patient and stakeholder perspective and to reach new and different audiences; (4) identifying new or different opportunities to share information about the study, to move beyond traditional models of dissemination; (5) involving national organizations, including the American Academy of Pain Medicine, the American Pain Society, the Society of General Internal Medicine, the American Academy of Family Physicians and patient advocacy organizations in dissemination of study findings, with similar efforts on

regional and local levels; and (6) working with PCORI through their venues for dissemination and translation.

12. DISSEMINATION

Potential for disseminating and implementing the results of this research in other settings. If our health system intervention of provider-targeted behavioral interventions are effective in preventing unsafe opioid prescribing, it will be critically important for dissemination and implementation (D&I) to occur rapidly and broadly. A potential boost to D&I in the case of our proposal is the widespread use and high market share of the Epic EHR. Despite that, barriers often compromise diffusion of research result into practice and diminish potential public health impact. With our Stakeholder Advisory Committee and other stakeholders, we will target multiple areas of dissemination. We will use the Expert Recommendations for Implementing Change (ERIC) recommended strategies to guide our D&I efforts (Table 2).

Table 2. ERIC Strategies for Dissemination and Implementation

ERIC Strategy	Activity
**Gathering information to prepare for Dissemination and Implementation**	**Assess Need and Readiness**<br>• We will incorporate queries on D&I and preferences regarding receipt of findings into our year 3 qualitative interviews with providers and patients. In this way, we will better understand the barriers and needs faced by providers and patients during acute pain episodes.<br>• To maximize D&I impact, we will work with stakeholders throughout the study and after completion to refine the assessment of needs and readiness
**Building Buy-in/Developing Relationships**	**Collaborate with Stakeholder Advisory Committee**<br>• Building relationships across multi-stakeholders will be a core goal of the Stakeholder Advisory Committee throughout the study.<br>• The Committee will provide consultation and feedback on outcomes, analyses, and interpretation to ensure needs and concerns are understood and considered<br>• Utilized Committee to monitor dissemination progress
**Educating/Influencing Stakeholders**	**Develop/Distribute Education Materials**<br>• Develop materials to distribute to providers in multiple formats (e.g., publications, presentations, internet, webinars)<br>• Work with national organizations such as including the American Academy of Pain Medicine, the American Pain Society, the Society of General Internal Medicine, the American Academy of Family Physicians and PCORI to disseminate findings
**Ensuring Quality Management**	**Work with key stakeholders to implement findings**<br>• Work with Stakeholder Advisory Committee to identify

	health systems, federal and private payors, health information technology organizations that can help develop implementation strategies and tools

Possible barriers to disseminating and implementing the results of this research in other settings and also describe any other study limitations that could have an impact on the usability of the findings. Even if proven effective, potential barriers to D&I of the provider-targeted interventions may arise at the health system, provider, and patient level. For the health system, there may be resistance to take steps to program the behavioral interventions into the Epic EHR or concern about restricting provider's autonomy. This may be mitigated by sharing high quality data about the impact of the intervention on outcomes and participant reactions as well as providing access to information and tools for implementing the intervention components. In addition, additional staff training and development, and access to our Epic EHR programming approach may be required prior to implementation. For providers, there may be resistance based on lack of awareness of study results, resistance to changing practice patterns, or concern the results do not apply to them or their patients. This may be mitigated by publishing in high impact journals with reach, disseminating via webinar, building a study website to inform providers, and disseminating results and recommendations through professional organizations. For patients, there may be concern implementation of such provider-targeted interventions might interfere with the doctor-patient relationship, impact provider autonomy, and perhaps lead to under-treatment of acute pain. To mitigate this, the research team, Stakeholder Advisory Committee, and health system must work closely with patients and caregivers to identify messages and means of communication to clearly articulate the benefits and potential risks of the provider-targeted interventions.

Making study results available to study participants. In close collaboration with the Stakeholder Advisory Committee and the study team will develop a full-color printed newsletter for distribution to our provider and consented patient participants and other entities that we feel the study information will be particularly relevant, such as health system administrators, payers, providers, and community organization. Our patient partners will be asked to provide input to ensure that the results are framed in ways that are relevant to patients and the messages are appropriately tailored to their customary communication styles. We will also include the newsletter on our implementation website and this web-based version will be updated with new information about subsequent intervention scaling efforts. The link to our website will be provided to all participants.

13. REFERENCES

1. Nahin, R.L., *Estimates of pain prevalence and severity in adults: United States, 2012.* J Pain, 2015. **16**(8): p. 769-80.
2. Medicine, I.o., *Relieving Pain in America: A Blueprint for Transforming Prevention, Care, Education, and Research*. 2011, Washington, DC: The National Academies Press.
3. Mafi, J.N., et al., *Worsening trends in the management and treatment of back pain.* JAMA Intern Med, 2013. **173**(17): p. 1573-81.
4. Deyo, R.A., M. Von Korff, and D. Duhrkoop, *Opioids for low back pain.* Bmj, 2015. **350**: p. g6380.
5. Mafi, J.N., et al., *Trends in the ambulatory management of headache: analysis of NAMCS and NHAMCS data 1999-2010.* J Gen Intern Med, 2015. **30**(5): p. 548-55.
6. Epstein, H., C. Hansen, and D. Thorson, *A protocol for addressing acute pain and prescribing opioids.* Minn Med, 2014. **97**(4): p. 47-51.
7. Zhang, W., et al., *OARSI recommendations for the management of hip and knee osteoarthritis, Part II: OARSI evidence-based, expert consensus guidelines.* Osteoarthritis Cartilage, 2008. **16**(2): p. 137-62.
8. Dowell, D., T.M. Haegerich, and R. Chou, *CDC Guideline for Prescribing Opioids for Chronic Pain--United States, 2016.* Jama, 2016. **315**(15): p. 1624-45.
9. Califf, R.M., J. Woodcock, and S. Ostroff, *A Proactive Response to Prescription Opioid Abuse.* N Engl J Med, 2016. **374**(15): p. 1480-5.
10. Volkow, N.D. and A.T. McLellan, *Opioid Abuse in Chronic Pain--Misconceptions and Mitigation Strategies.* N Engl J Med, 2016. **374**(13): p. 1253-63.
11. Hughes A, W.M., Lipari RN, et al., *Prescription drug use and misuse in the United States: Results from the 2015 National Survey on Drug Use and Health*. 2016, SAMSHA.
12. Hedegaard, H., L.H. Chen, and M. Warner, *Drug-poisoning deaths involving heroin: United States, 2000-2013.* NCHS Data Brief, 2015(190): p. 1-8.
13. Jones, C.M., et al., *Vital Signs: Demographic and Substance Use Trends Among Heroin Users - United States, 2002-2013.* MMWR Morb Mortal Wkly Rep, 2015. **64**(26): p. 719-25.
14. Compton, W.M., C.M. Jones, and G.T. Baldwin, *Relationship between Nonmedical Prescription-Opioid Use and Heroin Use.* N Engl J Med, 2016. **374**(2): p. 154-63.
15. Cicero, T.J. and M.S. Ellis, *Nonmedical Prescription-Opioid Use and Heroin Use.* N Engl J Med, 2016. **374**(13): p. 1295-6.
16. Cicero, T.J., et al., *The changing face of heroin use in the United States: a retrospective analysis of the past 50 years.* JAMA Psychiatry, 2014. **71**(7): p. 821-6.
17. Nuckols, T.K., et al., *Opioid prescribing: a systematic review and critical appraisal of guidelines for chronic pain.* Ann Intern Med, 2014. **160**(1): p. 38-47.
18. Cheatle, M.D. and C. Barker, *Improving opioid prescription practices and reducing patient risk in the primary care setting.* J Pain Res, 2014. **7**: p. 301-11.
19. Windmill, J., et al., *Interventions for the reduction of prescribed opioid use in chronic non-cancer pain.* Cochrane Database Syst Rev, 2013(9): p. Cd010323.
20. Chou, R., et al., *The effectiveness and risks of long-term opioid therapy for chronic pain: a systematic review for a National Institutes of Health Pathways to Prevention Workshop.* Ann Intern Med, 2015. **162**(4): p. 276-86.
21. Fox, T.R., et al., *A performance improvement prescribing guideline reduces opioid prescriptions for emergency department dental pain patients.* Ann Emerg Med, 2013. **62**(3): p. 237-40.
22. Penti, B., et al., *Novel peer review method for improving controlled substance prescribing in primary care.* J Opioid Manag, 2016. **12**(4): p. 269-79.

23. Grimshaw, J.M., et al., *Changing provider behavior: an overview of systematic reviews of interventions.* Med Care, 2001. **39**(8 Suppl 2): p. Ii2-45.
24. Bates, D.W., et al., *Ten Commandments for Effective Clinical Decision Support: Making the Practice of Evidence-based Medicine a Reality.* Journal of the American Medical Informatics Association, 2003. **10**(6): p. 523-530.
25. Embi, P.J. and A.C. Leonard, *Evaluating alert fatigue over time to EHR-based clinical trial alerts: findings from a randomized controlled study.* J Am Med Inform Assoc, 2012. **19**(e1): p. e145-8.
26. Emanuel, E.J., et al., *Using Behavioral Economics to Design Physician Incentives That Deliver High-Value Care.* Ann Intern Med, 2016. **164**(2): p. 114-9.
27. Patel, M.S. and K.G. Volpp, *Leveraging insights from behavioral economics to increase the value of health-care service provision.* J Gen Intern Med, 2012. **27**(11): p. 1544-7.
28. Loewenstein, G., T. Brennan, and K.G. Volpp, *Asymmetric paternalism to improve health behaviors.* Jama, 2007. **298**(20): p. 2415-7.
29. Loewenstein, G., D.A. Asch, and K.G. Volpp, *Behavioral economics holds potential to deliver better results for patients, insurers, and employers.* Health Aff (Millwood), 2013. **32**(7): p. 1244-50.
30. Navathe, A.S. and E.J. Emanuel, *Physician Peer Comparisons as a Nonfinancial Strategy to Improve the Value of Care.* Jama, 2016. **316**(17): p. 1759-1760.
31. Meeker, D., et al., *Nudging guideline-concordant antibiotic prescribing: a randomized clinical trial.* JAMA Intern Med, 2014. **174**(3): p. 425-31.
32. Meeker, D., et al., *Effect of Behavioral Interventions on Inappropriate Antibiotic Prescribing Among Primary Care Practices: A Randomized Clinical Trial.* Jama, 2016. **315**(6): p. 562-70.
33. Tannenbaum, D., et al., *Nudging physician prescription decisions by partitioning the order set: results of a vignette-based study.* J Gen Intern Med, 2015. **30**(3): p. 298-304.
34. Patel, M.S., et al., *Using default options within the electronic health record to increase the prescribing of generic-equivalent medications: a quasi-experimental study.* Ann Intern Med, 2014. **161**(10 Suppl): p. S44-52.
35. Keller, P.A., et al., *Enhanced active choice: A new method to motivate behavior change.* Journal of Consumer Psychology, 2011. **21**(4): p. 376-383.
36. Lerner, J.S. and P.E. Tetlock, *Accounting for the effects of accountability.* Psychol Bull, 1999. **125**(2): p. 255-75.
37. De Cremer, D. and M. Barker, *Accountability and cooperation in social dilemmas: The influence of others' reputational concerns.* Current Psychology, 2003. **22**(2): p. 155-163.
38. Milinski, M., D. Semmann, and H.J. Krambeck, *Reputation helps solve the 'tragedy of the commons'.* Nature, 2002. **415**(6870): p. 424-6.
39. Ayres, I., S. Raseman, and A. Shih, *Evidence from Two Large Field Experiments that Peer Comparison Feedback Can Reduce Residential Energy Usage.* Journal of Law, Economics, and Organization, 2013. **29**(5): p. 992-1022.
40. Ivers, N., et al., *Audit and feedback: effects on professional practice and healthcare outcomes.* Cochrane Database Syst Rev, 2012(6): p. Cd000259.
41. Hallsworth, M., et al., *Provision of social norm feedback to high prescribers of antibiotics in general practice: a pragmatic national randomised controlled trial.* Lancet, 2016. **387**(10029): p. 1743-52.
42. Waldo, S.W., et al., *Association between public reporting of outcomes with procedural management and mortality for patients with acute myocardial infarction.* J Am Coll Cardiol, 2015. **65**(11): p. 1119-26.
43. Keyes, K.M., et al., *Understanding the rural-urban differences in nonmedical prescription opioid use and abuse in the United States.* Am J Public Health, 2014. **104**(2): p. e52-9.

44. Kean, J., et al., *Comparative Responsiveness of the PROMIS Pain Interference Short Forms, Brief Pain Inventory, PEG, and SF-36 Bodily Pain Subscale.* Med Care, 2016. **54**(4): p. 414-21.
45. Krebs, E.E., et al., *Comparative responsiveness of pain outcome measures among primary care patients with musculoskeletal pain.* Med Care, 2010. **48**(11): p. 1007-14.
46. Krebs, E.E., et al., *Development and initial validation of the PEG, a three-item scale assessing pain intensity and interference.* J Gen Intern Med, 2009. **24**(6): p. 733-8.
47. Bohnert, A.S., et al., *A Detailed Exploration Into the Association of Prescribed Opioid Dosage and Overdose Deaths Among Patients With Chronic Pain.* Med Care, 2016. **54**(5): p. 435-41.
48. Bohnert, A.S., et al., *Association between opioid prescribing patterns and opioid overdose-related deaths.* Jama, 2011. **305**(13): p. 1315-21.
49. Park, T.W., et al., *Benzodiazepine prescribing patterns and deaths from drug overdose among US veterans receiving opioid analgesics: case-cohort study.* Bmj, 2015. **350**: p. h2698.
50. Sun, E.C., et al., *Incidence of and Risk Factors for Chronic Opioid Use Among Opioid-Naive Patients in the Postoperative Period.* JAMA Intern Med, 2016. **176**(9): p. 1286-93.
51. Alam, A., et al., *Long-term analgesic use after low-risk surgery: a retrospective cohort study.* Arch Intern Med, 2012. **172**(5): p. 425-30.
52. Gunst, R.F. and R.L. Mason, *Fractional factorial design.* Wiley Interdisciplinary Reviews: Computational Statistics, 2009. **1**(2): p. 234-244.
53. Mascha, E.J. and D.I. Sessler, *Equivalence and noninferiority testing in regression models and repeated-measures designs.* Anesth Analg, 2011. **112**(3): p. 678-87.
54. Adams, G., et al., *Patterns of intra-cluster correlation from primary care research to inform study design and analysis.* J Clin Epidemiol, 2004. **57**(8): p. 785-94.
55. Loudon, K., et al., *The PRECIS-2 tool: designing trials that are fit for purpose.* Bmj, 2015. **350**: p. h2147.
56. Broyles, L.M., et al., *Evaluation of a pilot training program in alcohol screening, brief intervention, and referral to treatment for nurses in inpatient settings.* J Addict Nurs, 2013. **24**(1): p. 8-19.
57. Broyles, L.M., et al., *A tailored curriculum of alcohol screening, brief intervention, and referral to treatment (SBIRT) for nurses in inpatient settings.* J Addict Nurs, 2013. **24**(3): p. 130-41.
58. Broyles, L.M., et al., *A qualitative study of anticipated barriers and facilitators to the implementation of nurse-delivered alcohol screening, brief intervention, and referral to treatment for hospitalized patients in a Veterans Affairs medical center.* Addict Sci Clin Pract, 2012. **7**: p. 7.
59. Broyles, L.M., et al., *Hospitalized patients' acceptability of nurse-delivered screening, brief intervention, and referral to treatment.* Alcohol Clin Exp Res, 2012. **36**(4): p. 725-31.
60. Childers, J.W., et al., *Teaching the teachers: faculty preparedness and evaluation of a retreat in screening, brief intervention, and referral to treatment.* Subst Abus, 2012. **33**(3): p. 272-7.
61. Depp, T.B., et al., *Risk factors associated with acute exacerbation of chronic obstructive pulmonary disease in HIV-infected and uninfected patients.* Aids, 2016. **30**(3): p. 455-63.
62. Gordon, A.J., et al., *Update in addiction medicine for the primary care clinician.* J Gen Intern Med, 2008. **23**(12): p. 2112-6.
63. Green, T.C., et al., *Patterns of drug use and abuse among aging adults with and without HIV: a latent class analysis of a US Veteran cohort.* Drug Alcohol Depend, 2010. **110**(3): p. 208-20.
64. Justice, A.C., et al., *Risk of mortality and physiologic injury evident with lower alcohol exposure among HIV infected compared with uninfected men.* Drug Alcohol Depend, 2016. **161**: p. 95-103.
65. Korthuis, P.T., et al., *Unhealthy alcohol and illicit drug use are associated with decreased quality of HIV care.* J Acquir Immune Defic Syndr, 2012. **61**(2): p. 171-8.

66. Korthuis, P.T., et al., *Quality of HIV Care and Mortality Rates in HIV-Infected Patients.* Clin Infect Dis, 2016. **62**(2): p. 233-9.
67. Kraemer, K.L., et al., *Alcohol problems and health care services use in human immunodeficiency virus (HIV)-infected and HIV-uninfected veterans.* Med Care, 2006. **44**(8 Suppl 2): p. S44-51.
68. McGinnis, K.A., et al., *Number of Drinks to "Feel a Buzz" by HIV Status and Viral Load in Men.* AIDS Behav, 2016. **20**(3): p. 504-11.
69. McGinnis, K.A., et al., *Comparison of AUDIT-C collected via electronic medical record and self-administered research survey in HIV infected and uninfected patients.* Drug Alcohol Depend, 2016. **168**: p. 196-202.
70. Rubio, D.M., et al., *Brief motivational enhancement intervention to prevent or reduce postpartum alcohol use: a single-blinded, randomized controlled effectiveness trial.* J Subst Abuse Treat, 2014. **46**(3): p. 382-9.
71. Saitz, R., et al., *Some medical inpatients with unhealthy alcohol use may benefit from brief intervention.* J Stud Alcohol Drugs, 2009. **70**(3): p. 426-35.
72. Saitz, R., et al., *Brief intervention for medical inpatients with unhealthy alcohol use: a randomized, controlled trial.* Ann Intern Med, 2007. **146**(3): p. 167-76.
73. Cochran, G., et al., *An Examination of Claims-based Predictors of Overdose from a Large Medicaid Program.* Med Care, 2016.
74. Cochran, G., et al., *Defining Nonmedical Use of Prescription Opioids Within Health Care Claims: A Systematic Review.* Subst Abus, 2015. **36**(2): p. 192-202.
75. Gordon, A., et al., *Treatment Quality for Buprenorphine Care: The Pot at the End of the Rainbow.* J Addict Med, 2016. **10**(3): p. 210-1.
76. Gordon, A.J., et al., *Patterns and Quality of Buprenorphine Opioid Agonist Treatment in a Large Medicaid Program.* J Addict Med, 2015. **9**(6): p. 470-7.
77. Lo-Ciganic, W.H., et al., *Association between trajectories of buprenorphine treatment and emergency department and in-patient utilization.* Addiction, 2016. **111**(5): p. 892-902.
78. Jamison, R.N., et al., *Substance misuse treatment for high-risk chronic pain patients on opioid therapy: a randomized trial.* Pain, 2010. **150**(3): p. 390-400.
79. Smith, S.M., et al., *Pain intensity rating training: results from an exploratory study of the ACTTION PROTECCT system.* Pain, 2016. **157**(5): p. 1056-64.
80. Taylor, A.M., et al., *Assessment of physical function and participation in chronic pain clinical trials: IMMPACT/OMERACT recommendations.* Pain, 2016. **157**(9): p. 1836-50.
81. Wasan, A.D., *Efficacy vs effectiveness and explanatory vs pragmatic: where is the balance point in pain medicine research?* Pain Med, 2014. **15**(4): p. 539-40.
82. Wasan, A.D., et al., *Iatrogenic addiction in patients treated for acute or subacute pain: a systematic review.* J Opioid Manag, 2006. **2**(1): p. 16-22.
83. Wasan, A.D., et al., *Neural correlates of chronic low back pain measured by arterial spin labeling.* Anesthesiology, 2011. **115**(2): p. 364-74.
84. Wasan, A.D., et al., *Psychiatric Comorbidity Is Associated Prospectively with Diminished Opioid Analgesia and Increased Opioid Misuse in Patients with Chronic Low Back Pain.* Anesthesiology, 2015. **123**(4): p. 861-72.
85. Wasan, A.D., et al., *Craving of prescription opioids in patients with chronic pain: a longitudinal outcomes trial.* J Pain, 2012. **13**(2): p. 146-54.
86. Powell, B.J., et al., *A refined compilation of implementation strategies: results from the Expert Recommendations for Implementing Change (ERIC) project.* Implement Sci, 2015. **10**: p. 21.
87. Campbell MJ, Walters SJ. *How to design, analyse and report cluster randomised trials in medicine and health related research.* 2014. Chichester: John Wiley & Sons, Ltd.

14. SUPPLEMENTS/APPENDICES

Appendix A: Poster-Flyer in participating clinics

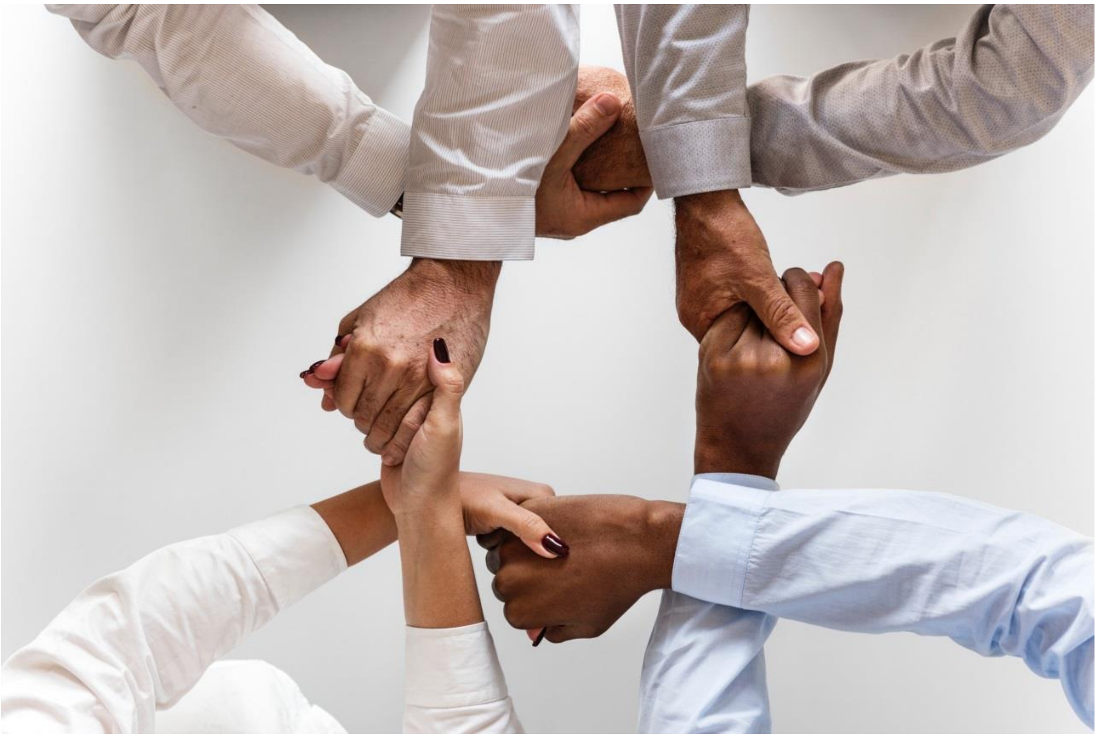

NOTICE TO OUR PATIENTS:

This primary care practice is taking part in a research project to promote evidence-based treatment for pain. If you have a clinic visit for pain between the dates of xx/xx/2018 and xx/xx/2019, you may be contacted by staff, via a phone call or a letter, to request permission for the project's evaluation team to contact you. Please speak with the clinic manager if you have questions. Thank you.

Appendix B: Information for Practices

This quality improvement project is focused on the prevention of unsafe opioid prescribing for patients with pain. The project is supported by the Patient-Centered Outcomes Research Institute (PCORI) and is coordinated by investigators at the University of Pittsburgh, in collaboration with UPMC, Geisinger Health System, and the University of Utah Health System. The project has received approval from the University of Pittsburgh Human Research Protection Office, which serves as the IRB of record for this project across the 3 health systems.

Key elements of the project include:

- 3 years duration, 2018-2021.
- Forty-eight primary care clinics across these 3 health systems will be randomized to 1 of 4 low-burden, electronic health record based interventions intended to promote evidence-based pain management. Providers will maintain full autonomy of management decisions.
- Patient outcomes will be extracted from the electronic health record by the project team and stored in de-identified format in the University of Pittsburgh Health Services Research Data Center for analysis.
- Participating clinics will be asked to provide some basic information about provider demographics (age, gender, years since completion of training), which will be linked to patient data in de-identified format for analytic purposes. Clinic and provider identifiers will not appear in reports.
- A subsample of patients with a qualifying initial clinic visit will be asked to complete 3 brief follow-up surveys at 1 month, 6 months, and 12 months. Participating clinics will be provided weekly with names of patients to contact and, with the aid of a brief script provided by the project team, request verbal permission for the project team to contact the patient. The clinic burden for this is expected to be approximately 1-2 brief patient calls per week from mid-2018 to mid-2019.
- Starting in mid-2019, a subsample of providers (approximately 1-3 per clinic) will be sent an e-mail to invite participation in a phone interview assess experience with the electronic health record intervention.
- For participation, practices will receive $1200 per provider in the practice.

Please feel free to contact [insert name] (Project Manager/Research Assistant) at [phone number] or [insert name] (Principal Investigator) at [phone number] for any questions. Thank you.

Appendix C: Information Sheet for Providers

Your primary care practice is participating in a quality improvement project focused on promotion of evidence-based management for pain in primary care. The project is supported by the Patient-Centered Outcomes Research Institute (PCORI) and is coordinated by investigators at the University of Pittsburgh, in collaboration with UPMC, Geisinger Health System, and the University of Utah Health System. The project has received approval from the University of Pittsburgh Human Research Protection Office, which serves as the IRB of record for this project across the 3 health systems.

Key elements of the project include:

- 3 years duration, 2018-2021.
- Forty-eight primary care clinics across these 3 health systems will be randomized to 1 of 4 low-burden, electronic health record based interventions intended to promote evidence-based pain management. Providers will maintain full autonomy of management decisions.
- Patient outcomes will be extracted from the electronic health record by the project team and stored in de-identified format in the University of Pittsburgh Health Services Research Data Center for analysis.
- Participating clinics will be asked to provide some basic information about provider demographics (age, gender, years since completion of training), which will be linked to patient data in de-identified format for analytic purposes. Clinic and provider identifiers will not appear in reports.
- A subsample of patients with a qualifying initial clinic visit will be asked to complete 3 brief follow-up surveys at 1 month, 6 months, and 12 months. Participating clinics will be provided weekly with names of patients to contact and, with the aid of a brief script provided by the project team, request verbal permission for the project team to contact the patient. The clinic burden for this is expected to be approximately 1-2 brief patient calls per week from mid-2018 to mid-2019.
- Starting in mid-2019, a random subsample of providers (approximately 1-3 per clinic) will be sent an e-mail to invite participation in a phone interview assess experience with the electronic health record intervention.

Please feel free to contact Jane Doe (Project Manager) at xxx-xxx-xxxx or Dr. Kevin Kraemer (Principal Investigator) at 412-692-4843 for any questions. Thank you.

Appendix D: Clinic Letter to Patients (subset of 642)

[date]

[name]
[address]

Dear [patient name]:

Our primary care practice is taking part in a research project focused on promoting evidence-based management for pain.

We are contacting you now because you had a recent clinic visit with a pain-related diagnosis. To see how you are doing following that visit, the project staff would like to do 3 very brief online or phone surveys at 1 month, 6 months, and 12 months after your initial visit. The survey questions will focus on pain, function and satisfaction with your care. Each survey will take less than 5 minutes online or by phone, and we will compensate you for your time.

Evaluation staff will contact you soon to find out if you are interested in participating and to answer any questions you may have. Or, if you prefer, you can visit the website below to learn more information and to provide your consent for the surveys. Participation is completely voluntary, so you may also call [insert phone number] and ask not to be contacted.

[insert website link]
[insert access code]

If you have any questions or do not have internet access, please contact [insert name] (Project Manager/ Research Assistant) at [phone number] or [insert name] (Principal Investigator) at [phone number].

If you have questions about participant rights, please contact [insert IRB contact information].

Thank you for considering this request. And as always, we appreciate the opportunity to provide you with high-quality, comprehensive care.

Sincerely,

[clinic director]

Appendix E: Clinic Phone Call Script to Patients (subset of 642)

[Greeting]

Hi Ms./Mr. [patient last name]. This is [clinic staff name] from the [name of practice].

Our primary care practice is taking part in a quality improvement project focused on promoting evidence-based management for pain. We are contacting you now because you had a recent clinic visit with a pain-related diagnosis. To see how you are doing following that visit, the evaluation staff for the project wish to do very brief online (web-based internet) or phone surveys at 1 month, 6 months, and 12 months following that initial visit. The survey questions will focus on pain, function, and satisfaction with care. Each survey will take less than 5 minutes online or by phone and you will be compensated for your time.

Evaluation staff will be contacting you in the near future to assess your interest in participating and to answer questions. Or, if you prefer, you can go to [website hyperlink] to learn more information and to provide consent for the surveys. As participation is completely voluntary, you may also call xxx-xxx-xxxx and ask to not be contacted.

Do you have any questions?

Thank you for your consideration of this request. As always, we thank you for the privilege of providing high-quality, comprehensive care to you.

Appendix F: Phone Verbal Consent for Patients (subset of 642)

[Greeting] Hello, Ms./Mr. [patient last name], my name is [staff member's name].

Thank you for taking the time to talk today. I wish to tell you about a research project that is ongoing in your primary care practice. The project promotes safe and appropriate care for patients with pain. You were contacted because you had a recent doctor's office visit for pain. To see how you are doing after that visit, we wish to do very brief online (web-based) or phone surveys. You will complete the survey at 1 month, 6 months, and 12 months following the initial visit. The survey questions focus on pain, function, and satisfaction with care. Each survey will take less than 5 minutes online or by phone. You will receive $15.00 for each completed survey. The maximum amount you will receive is $45.00 if all 3 surveys are completed.

Here is some other important information about the project and your potential participation:

- The project is supported by the Patient-Centered Outcomes Research Institute (PCORI). It is managed by a team at the University of Pittsburgh. The project also includes the University of Pittsburgh Medical Center (UPMC), Geisinger Health System, and the University of Utah Health System.
- If you agree to participate, you will be sent a link to the online survey site. You will receive e-mail reminders when a new survey needs to be done. We can arrange to have the surveys completed by phone if you are not comfortable with e-mail and an online survey.
- The survey results will be sent to the University of Pittsburgh for analysis. All identifying information will be removed. The results will not be linked to your name. They will be linked to a random number.
- There is a minimal risk of loss of privacy. The project staff will take every step to protect your identity and the privacy of your information.
- You will not directly benefit from participation. You may receive indirect benefit by helping improve our understanding of pain care in primary care.
- Your $15.00 payment will be sent after each completed survey.
- Your participation in this project is voluntary. Your participation will not affect the care you receive from your doctor.

Do you have any questions?

Are you interested in participating?

[If yes] Thank you, we really appreciate it. Let's take some additional information so our team can contact you to schedule the interview.

[If no] Thank you, we appreciate your willingness to learn about the project.

Please contact [insert name] (Project Manager/Research Assistant) at [phone number] or [insert name] (Principal Investigator) at [phone number] for any questions.

If you have questions about participant rights, please contact [IRB contact information].

Additional Payment Information:

Due to [insert institution name] policy and federal rules, we may have to collect additional information to pay you for participation. Please provide your phone number below so a member of our project staff can contact you. Project staff will call you within one week to collect the information.

Phone number: [text box]

Appendix G: Online Web Consent for Patients (subset of 642)

Thank you for visiting this website today. We wish to tell you about a research project that is ongoing in your primary care practice. The project promotes safe and appropriate care for patients with pain. You were contacted because you had a recent doctor's office visit for pain. To see how you are doing after that visit, we wish to do very brief online (web-based) or phone surveys. You will complete the survey at 1 month, 6 months, and 12 months following the initial visit. The survey questions focus on pain, function, and satisfaction with care. Each survey will take less than 5 minutes online or by phone. You will receive $15.00 for each completed survey. The maximum amount you will receive is $45.00 if all 3 surveys are completed.

Here is some important information about the project and your participation:

- The project is supported by the Patient-Centered Outcomes Research Institute (PCORI). It is managed by a team at the University of Pittsburgh. The project also includes the University of Pittsburgh Medical Center (UPMC), Geisinger Health System, and the University of Utah Health System.
- If you agree to participate, you will be sent a link to the online survey site. You will receive e-mail reminders when a new survey needs to be done. We can arrange to have the surveys completed by phone if you are not comfortable with e-mail and an online survey.
- The survey results will be sent to the University of Pittsburgh for analysis. All identifying information will be removed. The results will not be linked to your name. They will be linked to a random number.
- There is a minimal risk of loss of privacy. The project staff will take every step to protect your identity and the privacy of your information.
- You will not directly benefit from participation. You may receive indirect benefit by helping improve our understanding of pain care in primary care.
- Your $15.00 payment will be sent after each completed survey.
- Your participation in this project is voluntary. Your participation will not affect the care you receive from your doctor.

Do you have any questions? ["Yes" and "No" button options to click]

Are you interested in participating? ["Yes" and "No" button options to click]

[If yes] Thank you, we really appreciate it. Let's take some additional information so our team can contact you to schedule the interview.

[If no] Thank you, we appreciate your willingness to learn about the project.

Please contact [insert name] (Project Manager/Research Assistant) at [phone number] or [insert name] (Principal Investigator) at [phone number] for any questions.

If you have questions about participant rights, please contact [IRB contact information].

Additional Payment Information:

Due to [insert institution name] policy and federal rules, we may have to collect additional information to pay you for participation. Please provide your phone number below so a member of our project staff can contact you. Project staff will call you within one week to collect the information.

Phone number: [text box]

Appendix H: Recruitment E-mail to Providers (random 100)

Send To: [provider's email address]
Subject: Invitation to Participate in Interview – PCORI Pain Project

Dear Dr. [provider's name]:

Since 2018, [institution name] and [clinic name], [insert for Geisinger: in collaboration with the University of Pittsburgh and University of Utah Health; insert for Pittsburgh: in collaboration with Geisinger Health System and University of Utah Health; insert for Utah: in collaboration with the University of Pittsburgh and Geisinger health system], have been participating in a research project to promote evidence-based management for acute pain. The research interventions included an EHR Best Practice Alert regarding pain management and opioid prescribing, and, depending on your practice site, monthly e-mails containing opioid prescribing rates for opioid naïve patients.

You were randomly chosen to participate in a telephone interview about your thoughts and experiences with the provider-targeted research interventions. We would also like to gather your insights on the current landscape of opioid prescribing and other, similar interventions that you may have experienced. The information gathered will be crucial to help determine whether these interventions should be disseminated to other primary care practices.

- The interview will be conducted by a project staff member at the University of Pittsburgh.
- The length of the interview is expected to be 20 to 30 minutes but may be longer depending on the amount of information you have to share.
- Your audio-recorded answers will be transcribed, de-identified, and then categorized.
- The evaluation of your comments along with those from other volunteers will allow us to identify broad topics that are important.
- A study of this information may allow us to make recommendations about better ways to promote evidence-based pain management practices in primary care settings.
- The only risk associated with participating is a small risk of loss of privacy, so it is best to schedule the interview when you are in a quiet, private place.
- The audio recordings will remain at the University of Pittsburgh and will not be linked with your name, address, or other contact information.

We greatly appreciate your consideration of this request. If you are interested in participating in the interview please consent by clicking on the following link: [website hyperlink].

Once you complete the consent, you will be contacted by a project staff member to arrange the interview. Participants will be compensated $50 for their time and should expect to receive the compensation within two weeks of the completed interview.

Please feel free to contact [insert name] (Project Manager/Research Assistant) at [phone number] or [insert name] (Principal Investigator) at [phone number] for any questions.

If you have questions about participant rights, please contact [IRB contact information].

Sincerely,

[signature]	[signature]
[primary care network director]	[principal investigator]

Appendix I: Phone Verbal Consent for Providers (random 100)

[greeting]

Hello, [provider's name]. My name is [name]. Your primary care practice has been participating in a research project to promote evidence-based management for pain. You have been randomly selected, along with several other providers from each of the 48 participating practices, to participate in a phone interview. The purpose of the interview is to assess your experience with the provider-targeted interventions of the project. Depending on your practice site, these interventions included electronic health record alerts regarding pain management and opioid prescribing and/or e-mailed feedback on opioid prescribing rates in opioid naïve patients.

The interview will be conducted by a project staff member at the University of Pittsburgh. The length of the interview is expected to be 20 to 30 minutes but may be longer depending on the amount of information you have to share. Your audio-recorded answers will be transcribed and categorized. The evaluation of your comments along with those from other volunteers will allow us to identify broad topics that are important. A study of this information may allow us to make recommendations about better ways to promote evidence-based pain management practices in primary care settings. The information gathered will be crucial to help determine whether these interventions should be disseminated to other primary care practices.

You will not directly benefit from participation. You may receive indirect benefit by helping improve our understanding of pain care in primary care. There is a minimal risk of loss of privacy. In order to minimize this risk it is best to schedule the interview when you are in a quiet, private place. The project staff will take every step to protect your identity and the privacy of your information. The audio recordings will remain at the University of Pittsburgh and will not be linked with your name, address, or other contact information. Your participation this interview is entirely voluntary.
You will be compensated $50 for participating in the phone interview. You will receive the compensation within one to two weeks of the completed interview.

Do you have any questions?

Are you interested in participating?

[If no] Thank you, we appreciate your willingness to hear about the project.

[If yes] Thank you, we really appreciate it. Let me take some additional contact information so our team can contact you to schedule the interview. [Complete form with the following fields: name, preferred phone number, preferred email address, preferred contact date(s) and time(s)] A project staff member from the University of Pittsburgh will contact you during your preferred date/time to schedule the interview; please be aware that you will receive a call from [insert phoned number].

Please contact [insert name] (Project Manager/Research Assistant) at [phone number] or [insert name] (Principal Investigator) at [phone number] for any questions.

If you have questions about participant rights, please contact [IRB contact information].

In addition to the investigator listed and their research staff, the following individuals may have access to your information related to your participation in this research study:

- Authorized representatives of the study sponsor and the University of Pittsburgh Office of Research Protections may review your identifiable research information for purposes of monitoring the conduct of this research study.
- Participating sites in this multi-site study for purposes of data analysis or other investigators conducting future research; however, this information will be shared in a de-identified manner (i.e., without identifiers).

Additional Payment Information:
Due to [insert institution name] policy and federal rules, we have to collect additional information to pay you for participation. Project staff may also collect additional required payment information at the time of your interview.
[Complete form with: street address, city, state, zip code]

Appendix J: Online Web Consent for Providers (random 100)

Thank you for visiting this website today. Your primary care practice has been participating in a research project to promote evidence-based management for pain. You have been randomly selected, along with several other providers from each of the 48 participating practices, to participate in a phone interview. The purpose of the interview is to assess your experience with the provider-targeted interventions of the project. Depending on your practice site, these interventions included electronic health record alerts regarding pain management and opioid prescribing and/or e-mailed feedback on opioid prescribing rates in opioid naïve patients.

The interview will be conducted by a project staff member at the University of Pittsburgh. The length of the interview is expected to be 20 to 30 minutes but may be longer depending on the amount of information you have to share. Your audio-recorded answers will be transcribed and categorized. The evaluation of your comments along with those from other volunteers will allow us to identify broad topics that are important. A study of this information may allow us to make recommendations about better ways to promote evidence-based pain management practices in primary care settings. The information gathered will be crucial to help determine whether these interventions should be disseminated to other primary care practices.

You will not directly benefit from participation. You may receive indirect benefit by helping improve our understanding of pain care in primary care. There is a minimal risk of loss of privacy. In order to minimize this risk it is best to schedule the interview when you are in a quiet, private place. The project staff will take every step to protect your identity and the privacy of your information. The audio recordings will remain at the University of Pittsburgh and will not be linked with your name, address, or other contact information. Your participation this interview is entirely voluntary.
You will be compensated $50 for participating in the phone interview. You will receive the compensation within one to two weeks of the completed interview.

Do you have any questions? [“Yes” and “No” button options to click]

Are you interested in participating? [“Yes” and “No” button options to click]

[If no] Thank you, we appreciate your willingness to hear about the project.

[If yes] Thank you, we really appreciate it. Let me take some additional contact information so our team can contact you to schedule the interview. A project staff member from the University of Pittsburgh will contact you within two weeks or during your preferred date/time to schedule the interview; please be aware that you will receive a call from [insert phone number].

[Complete form with the following fields: preferred phone number, preferred email address, preferred contact date(s) and time(s)]

Please contact [insert name] (Project Manager) at [phone number] or [insert name] (Principal Investigator) at [phone number] for any questions.

If you have questions about participant rights, please contact [IRB contact information].

In addition to the investigator listed and their research staff, the following individuals may have access to your information related to your participation in this research study:

- Authorized representatives of the study sponsor and the University of Pittsburgh Office of Research Protections may review your identifiable research information for purposes of monitoring the conduct of this research study.
- Participating sites in this multi-site study for purposes of data analysis or other investigators conducting future research; however, this information will be shared in a de-identified manner (i.e., without identifiers).

Additional Payment Information:
Due to [insert institution name] policy and federal rules, we have to collect additional information to pay you for participation; please complete the form below. Project staff may also collect additional required payment information at the time of your interview.

[Complete form with: street address, city, state, zip code]

Appendix K: EHR Data Extraction Computable Phenotype

Inclusion criteria (must meet each criteria):

- Age ≥ 18 years old
- Established outpatient for at least 12 months in the specific system (UPMC, Geisinger, or Utah) before the qualifying diagnosis visit
- Index outpatient clinic visit (primary care) for musculoskeletal pain and/or headache. Eligible ICD-10-CM diagnostic codes:
    - G43.00x, G43.1x, G43.4, G43.8, G44.0x, G44.2x, G44.1 (exclude any of these that use the .x1 or .xx9, indicating intractable headache)
    - G89.1
    - M15.xxx, M16.xxx, M17.xxx, M18.xxx, M19.xxx, M22.xxx, M23.xxx, M24.xxx, M25.xxx, M45.xxx, M46.xxx, M47.1xx, M47.2x, M47.81x, M89x, M50.xxx, M51.xxx, M53.xxx, M54.xxx, M65.xxx, M66.xxx, M67.xxx, M70.xxx, M71.xxx, M72.xxx (exclude M72.6), M75.xxx, M76.xxx, M77.xxx, M79.6xx, M94.xxx
    - R51, R52
    - S14.xx, S16.xxx, S19.9xx, S23.xxx, S24.xxx, S30.0xx, S30.810, S30.91XA, S40.xxx, S43.xxx, S50.xxx, S53.xxx, S59.9xx, S60.xxx, S63.xxx, S69.9xx, S70.xxx, S73.xxx, S79.9xx, S80.xxx, S83.xxx, S89.8x, S89.9x, S90.xxx, S93.xxx, S99.8. S99.9
    - [NOTE: I would also like to do a restricted look limited to the diagnoses of M54.2 Neck Pain, M54.5 low back pain, M54.6 thoracic pain, M79.6 pain in limb/hand/foot/fingers/toes, and R51 Headache]

Exclusion criteria (any will exclude patient):

- Cancer diagnosis on chart in past 12 months (includes ICD-10-CM in the Cxx.xxx and Dxx.xxx series; excluding non-melanoma skin cancer C44.xxx)
- Receipt of opioid prescription (oxycodone, hydrocodone, morphine, hydromorphone, oxymorphone, fentanyl, codeine, tramadol, methadone) within 12 months of index outpatient visit

Appendix L: Patient Subset (N=642) – Follow-Up Survey

Questions 1 through 3 are the PEG Screening Tool and question 4 is a satisfaction question.

1. What number best describes your pain on average in the past week:

0 1 2 3 4 5 6 7 8 9 10

No pain — Pain as bad as you can imagine

2. What number best describes how, during the past week, pain has interfered with your enjoyment of life?

0 1 2 3 4 5 6 7 8 9 10

Does not interfere — Completely interferes

3. What number best describes how, during the past week, pain has interfered with your general activity?

0 1 2 3 4 5 6 7 8 9 10

Does not interfere — Completely interferes

4. For the past 30 days, how *satisfied* are you with the management of your pain by your primary care practice?

0 1 2 3 4 5 6 7 8 9 10

Not at all satisfied — Completely satisfied

Appendix M: Milestones/Timetable

	Milestone Name	Description	Projected Completion Date
**A**	Project Start Date	**-**	**2/1/2018**
B1	Establish Study Advisory Committee	Identify members of the Study Advisory Committee (SAC). Document the role of each research partner, including patient, caregiver and other stakeholder partners, at each stage in the research process. Submit to PCORI roster of all Advisory Committee members, including names, titles, and roles on the project as well as financial compensation associated with participation in the project.	3/2//2018
B2	PCORI Kick-Off Call	Convene PCORI kick-off call with full project team.	3/2/2018
B3	Bi-Annual In-Person Meeting	Convene bi-annual in-person meeting with SAC, DSMB and Steering Committee. Please include PCORI staff in virtual invite. Meeting minutes/summary should be submitted along with progress report	3/31/2018
B4	Finalize and submit study protocol in PCORI Online	Finalize and submit to PCORI research protocol. Please refer to PCORI Methodology Standards for required elements of study protocol.	4/30/2018
B5	ClinicalTrials.gov Registration	Register study with ClinicalTrials.gov. Study Identification Number and primary completion date must be sent to PCORI. List PCORI as collaborator.	4/30/2018
B6	Submit updated Recruitment Plan in PCORI Online.	Elements in the recruitment plan should, at a minimum, include the following:<br>a. Timeline<br>b. Total target sample size for primary analysis<br>c. Name and # study sites<br>d. Historical patient volume and estimated eligible N across study sites<br>e. Estimated yield/consent<br>f. Estimated lost to follow up/attrition<br>g. Estimated monthly enrollment	4/30/2018
B7	Study Governance Plan	Establish a study governance plan and submit outline of governance and committee structure to PCORI.	5/31/2018
B8	Submit IRB approval in PCORI Online	Obtain IRB Approval for all study sites and send approval letter plus materials submitted to IRB to PCORI.	5/31/2018
B9	Clinic Site Recruitment	Complete primary care clinic site recruitment (n= 24 UPMC practices, 12 Geisinger practices, 12 Utah Health Care practices)	5/31/2018
B10	SAC Teleconference	Convene teleconference meeting with SAC; Meeting minutes/summary should be submitted along with progress report	6/30/2018
B11	Epic Programming	Complete Epic electronic health record programming, including acute pain opioid prescribing guideline, and free text justification field when prescribing opioids.	6/30/2018
B12	Submit updated Engagement Plan in PCORI Online.	Submit to PCORI an updated Engagement Plan. Elements of the updated Engagement Plan should include:<br>a. Update roster of committee/panel members with short bios<br>b. A Patient and/or Stakeholder Advisory Panel(s) or Committee(s) Governance Schematic<br>c. Planned training for patients and other stakeholder partners on the research process<br>d. Proposed Meeting Schedule<br>e. Tasks or opportunities wherein patients and/or stakeholders will have input via consultation, collaboration or leadership<br>f. Efforts to Evaluate/Assess Engagement	6/30/2018

B13	Submit Data Safety and Monitoring Plan to PCORI	Please refer to the PCORI Policy on Data Safety and Monitoring Plans for PCORI-Funded Research here: http://www.pcori.org/sites/default/files/PCORI-Policy-Data-Safety-Monitoring-Plans.pdf	6/30/2018
B14	Begin Patient Recruitment and Enrollment	Initiate patient recruitment across UPMC, Geisinger, and Utah Health systems (n=10,936 patients) (n=48 primary care clinics); From this point forward, submit monthly enrollment update to PCORI to include cumulative and interval recruitment, accrual, and retention for the overall study (e.g. number eligible/approached/consented/enrolled, retained). Discuss due dates for monthly reports with your Program Officer. Notify your Program Officer upon enrollment of the first participant.	7/31/2018
**B**	**Report Submission**	**Submit Progress Report, Using Current Interim Progress Report Template**	**8/1/2018**
C1	SAC Teleconference	Convene teleconference meeting with SAC. Meeting minutes/summary should be submitted along with progress report	9/30/2018
C2	Patient Recruitment	25% of patient participants enrolled in study.	10/31/2018
C3	1-Month Follow-up Telephone Survey	Complete 25% of 1 month follow-up telephone survey on patient reported outcomes of pain and function (n=500)	12/312018
C4	Patient Recruitment	50% of patient participants enrolled in study.	1/31/2019
C5	SAC Teleconference	Convene teleconference meeting with SAC	1/31/2019
C6	Bi-Annual In-Person Meeting	Convene bi-annual in-person meeting with SAC, DSMB and Steering Committee. Please include PCORI staff in virtual invite.	1/31/2019
**C**	**Report Submission**	**Submit Progress Report, Using Current Interim Progress Report Template**	**1/31/2019**
D1	1-Month Follow-up Telephone Survey	Complete 50% of 1 month follow-up telephone survey on patient reported outcomes of pain and function (n=1000)	3/31/2019
D2	IRB Annual Review	Submit IRB annual review approval letter to PCORI	4/1/2019
D3	Patient Recruitment	75% of patient participants enrolled in study.	4/30/2019
D4	SAC Teleconference	Convene teleconference meeting with SAC. Meeting minutes/summary should be submitted along with progress report	5/1/2019
D5	6-MonthFollow-up Telephone Survey	Complete 25% of 6 month follow-up telephone survey on patient reported outcomes of pain and function	5/31/2019
D6	1-Month Follow-up Telephone Survey	Complete 75% of 1 month follow-up telephone survey on patient reported outcomes of pain and function (n=1500)	6/30/2019
D7	Patient Recruitment	100% of patient participants enrolled in study.	7/31/2019
D8	Bi-Annual In-Person Meeting	Convene bi-annual in-person meeting with SAC, DSMB and Steering Committee. Please include PCORI staff in virtual invite. Meeting minutes/summary should be submitted along with progress report	7/31/2019
**D**	**Report Submission**	**Submit Progress Report, Using Current Interim Progress Report Template**	**10/31/2019**
E1	6-MonthFollow-up Telephone Survey	Complete 50% of 6 month follow-up telephone survey on patient reported outcomes of pain and function	1/31/2020
E2	1-Month Follow-up Telephone Survey	Complete 100% of 1 month follow-up telephone survey on patient reported outcomes of pain and function (n=2000)	1/31/2020
E3	SAC Teleconference	Convene teleconference meeting with SAC. Meeting minutes/summary should be submitted along with progress report	1/31/2020
E4	6-Month Follow-up Telephone Survey	Complete 75% of 6 month follow-up telephone survey on patient reported outcomes of pain and function	1/31/2020
E5	12-Month Follow-up Telephone Survey	Complete 25% of 12 month follow-up telephone survey on patient reported outcomes of pain and function	2/29/2020
E6	Qualitative Interviews	Begin qualitative telephone interviews of participating providers (n=100) attitutes and perceptions on how the interventions were perceived.	3/31/2020
E7	Bi-Annual In-Person Meeting	Convene bi-annual in-person meeting with SAC, DSMB and Steering Committee. Please include PCORI staff in virtual invite. Meeting minutes/summary should be submitted along with progress report	3/31/2020

**E**	**Report Submission**	**Submit Progress Report, Using Current Interim Progress Report Template**	**3/31/2020**
F1	12-Month Follow-up Telephone Survey	Complete 50% of 12 month follow-up telephone survey on patient reported outcomes of pain and function	4/30/2020
F2	6-MonthFollow-up Telephone Survey	Complete 100% of 6 month follow-up telephone survey on patient reported outcomes of pain and function	5/31/2020
F3	Qualitative Interviews	Complete qualitative telephone interviews of participating providers (n=100) attitutes and perceptions on how the interventions were perceived.	5/31/2020
F4	IRB Annual Review	Submit IRB annual review approval letter to PCORI	7/30/2020
F5	SAC Teleconference	Convene teleconference meeting with SAC. Meeting minutes/summary should be submitted along with progress report	7/30/2020
F6	12-Month Follow-up Telephone Survey	Complete 75% of 12 month follow-up telephone survey on patient reported outcomes of pain and function	8/31/2020
F7	Bi-Annual In-Person Meeting	Convene bi-annual in-person meeting with SAC, DSMB and Steering Committee. Please include PCORI staff in virtual invite. Meeting minutes/summary should be submitted along with progress report	8/31/2020
**F**	**Report Submission**	**Submit Progress Report, Using Current Interim Progress Report Template**	**10/31/2020**
G1	12-Month Follow-up Telephone Survey	Complete 100% of 12 month follow-up telephone survey on patient reported outcomes of pain and function	11/30/2020
**G**	**Primary Completion Date**	**A Primary Research Completion Date must be provided when registering the study in Clinicaltrials.gov. For studies that are not clinical trials or observational studies registered on ClinicalTrials.gov, the Awardee and PCORI shall agree on a primary completion date as a milestone that precedes the agreed-upon date to submit a Draft Final Research Report.**	**1/30/2021**
H1	Finalize Codebook	Finalize codebook for provider and patient interviews.	1/30/2021
H2	SAC Teleconference	Convene teleconference meeting with SAC. Meeting minutes/summary should be submitted along with progress report	1/31/2021
H3	Dissimination Plan Finalized	Develop plan for publications and dissemination in collaboration with patient partners and stakeholders that make findings available and useful to patients and providers in making health care decisions.	2/28/2021
H4	Manuscript Preparation and Submission	Prepare study manuscripts for submission and presentation of study findings.	2/28/2021
H5	Final Analyses	Complete final analyses for all study outcomes	4/30/2021
H6	Stakeholder Advisory Committee Meeting	Convene sixth meeting of the SAC. Meeting minutes/summary should be submitted along with progress report	5/31/2021
H7	Bi-Annual In-Person Meeting	Convene bi-annual in-person meeting with SAC, DSMB and Steering Committee. Please include PCORI staff in virtual invite. Meeting minutes/summary should be submitted along with progress report	8/31/2021
**H**	**Final Progress Report**	**Submit Final Progress Report, Using Final Progress Report Template**	**8/31/2021**
**I**	**Research Project Period End Date**	**Research Project Period End Date**	**10/31/2021**
**J**	**Results submitted to ClinicalTrials.gov**	Awardee ensures results are submitted to ClinicalTrials.gov. For ClinicalTrials.gov, the generated tables are a required section in the Draft Final Research Report. Results must be submitted no later than 30 days before Draft Final Research Report Submission Milestone to provide time for ClincialTrials.gov to conduct quality checks.	**10/31/2021**
**K**	**Draft Final Research Report Submission**	Submit Draft Final Research Report according to instructions found at: *http://www.pcori.org/awardee-resources*<br><br>*All Draft Final Research Reports must be submitted no later than 30 days from when results are posted to clinicaltrials.gov or other applicable website. Refer to Contract.	**12/1/2021**

**L**	**Final Research Report**	Upon receipt of written summary, and as applicable, PI will make revisions and submit revised Draft Final Research Report for acceptance within 90 days.	**9/1/2022**
**M**	**Approval / sign off of the Lay Abstract**	Sign off must be no later than 90 days beyond the date PCORI accepts the final research report	**See Description**
**N**	**Contract Term Date**	**Contract Term Date**	**11/30/2022**
**O**	**Final Expenditure Report**	**Submit Final Expenditure Report (See Contract for Instructions)**	**Within 90 Days from Contract Term Date**
**P**	**Notification of Public Acceptance**	**See Contract for Instructions**	**Within 30 Days of Acceptance**
