# Supplementary material for: Effect of Different Interventions to Help Primary Care Clinicians Avoid Unsafe Opioid Prescribing in Opioid-Naive Patients With Acute Noncancer Pain: A Cluster Randomized Clinical Trial
Source: JAMA Health Forum. 2022 Jul 29;3(7):e222263. doi: 10.1001/jamahealthforum.2022.2263 (PMC9338412; doi:10.1001/jamahealthforum.2022.2263)
Supplement: Supplement 2. — Statistical Analysis Plan [file jamahealthforum-e222263-s002.pdf]

Appendix Describing Changes to Original Statistical Analysis Plan for Primary Analyses

In the Research Plan submitted to PCORI, attached as a supplement to this manuscript, the primary analysis for our primary outcome was described as follows:

"The primary outcome, initial opioid prescription, is expected to decrease in all four intervention groups and our goal is to detect the difference in change in the outcome across the four groups. For our primary analysis, we will use logistic regression to compare the initial opioid prescription after the intervention initiation across the four groups, with baseline opioid prescription rate at each clinic (during 1 year before the intervention) and other important clinical characteristics as covariates. To investigate further the effect of the interventions given the changing outcome rates in the background, we will use piecewise mixed effect logistic regression with a knot at month 0 (intervention start date). Fixed effects for this model will include an intervention group indicator (guideline (usual care) as reference group, a dummy variable for opioid justification, and another dummy variable for provider comparison), time (in month), intervention period indicator (1 after intervention starts, 0 before intervention starts) time since intervention start (in month), interaction between [opioid justification and (intervention period indicator)], interaction between [provider comparison and (intervention period indicator)], interaction between [opioid justification and (time since intervention start)], interaction between [provider comparison and (time since intervention start)], interaction between [opioid justification and provider comparison and (time since intervention start)], stratification factors, and other clinical covariates as fixed effects. We will include random effects for providers, clinics, and health systems to allow for clustering effect within each provider, clinic and health systems. If any of the four interaction terms (intervention

period indicator × opioid justification, intervention period indicator × provider comparison, time since intervention start × opioid justification, time since intervention start × provider comparison) turns out to be significant, we can infer that the rate of initial opioid prescription either had sudden drop or decreased faster in the corresponding intervention group than the guideline (usual care) group."

Unfortunately, there were several practical issues during the data extraction and analysis phase that required changes to the proposed statistical model. The first problem relates to the intended use of data from 1 year prior to the intervention period. Despite several meetings between the study PI, study statistician, and database personnel responsible for identifying and extracting participants analogous to our study participants in the year prior to study activation, queries repeatedly returned data that were implausible (specifically, the number of participants per-month meeting study eligibility criteria were dramatically different at each study practice in the pre-intervention period than during the study period). After several efforts to isolate the problem did not reveal a clear reason for the discrepancy, the study team agreed that the pre-intervention data must not be capturing the same population as the intervention-period data, and it was better to use data from start of the intervention period only (which could be more easily verified) rather than pooling 2 potentially disparate sources for data analysis. Unfortunately, as the reviewers noted, the lack of pre-intervention data meant that we could not compute the "baseline opioid prescription rate" for each practice in the same population as our study target population. This also rendered all terms above with an "intervention period indicator" unusable; while prior studies (such as Meeker et al) used pre-intervention data in their analyses to estimate treatment effect, we would not be able to do this.

The second problem relates to the intended use of three-level nested random effects “for providers, clinics, and health systems” – with a large number of clinicians that saw a relatively small number of study-eligible participants and the lower-than-anticipated incidence of the primary outcome, a model with this hierarchical structure of random effects would not converge. We opted to use a simpler model with random effects for clinic and health system, omitting the “provider” level from the random effect mentioned in the SAP. Note that with the design randomizing at the clinic level, it is not strictly required to account for clustering at the level of a smaller unit – using a random effect for clinic matches the study design.

The combined effects of these issues were such that we had to strike any variable from the analysis which relied on “pre-intervention” data: that eliminated the covariate for each clinic’s “baseline opioid prescribing rate” as well as all “intervention period indicator” terms in the model – as well as the random effect for “provider” in the specified SAP. As a result, we were forced to fit a simpler statistical model than the model described above for our primary analyses testing the treatment effect of each assignment on opioid prescribing at the index visit. The primary analytic model used to estimate the treatment effect of each assignment in our submitted manuscript is a mixed-effects logistic regression model with a fixed effect for practice assignment to the Justification intervention, a fixed effect for practice assignment to the Comparison intervention, a fixed effect for practice geography (urban vs. rural), a fixed effect for study month, with random effects for study practice and health system. We report results for the "main effects only” model which includes a yes/no indicator for each of the Comparison and Justification interventions, but not a separate indicator for receiving both interventions.

In addition to the aforementioned changes in the primary analytic model, the reader may also note some differences in our intended secondary outcomes versus what we are able to report in the present manuscript. The reason for is principally the large amounts of missing data in many fields of the PRESCRIBING table in the Common Data Model used for extraction of our study outcomes. While we have the order date and the medication name available for each prescription in the table, fields such as dose, frequency, and duration had substantial amounts of missingness (over 50% for several key fields), making it impossible to accurately determine several of our intended secondary outcomes such as whether patients received high-dose opioid therapy. We were able to compute one secondary outcome of chronic opioid therapy (defined as having three or more prescriptions within a three month period) and another secondary outcome of concurrent opioid/benzodiazepine use (defined as having an opioid prescription and a benzodiazepine prescription within 30 days of one another), but could not fully execute our originally planned secondary outcomes exactly as originally defined.
