# Supplementary material for: Effect of Different Interventions to Help Primary Care Clinicians Avoid Unsafe Opioid Prescribing in Opioid-Naive Patients With Acute Noncancer Pain: A Cluster Randomized Clinical Trial
Source: JAMA Health Forum. 2022 Jul 29;3(7):e222263. doi: 10.1001/jamahealthforum.2022.2263 (PMC9338412; doi:10.1001/jamahealthforum.2022.2263)
Supplement: Supplement 4. — Data Sharing Statement [file jamahealthforum-e222263-s004.pdf]

Data Sharing Statement

Kraemer. Effect of Different Interventions to Help Primary Care Clinicians Avoid Unsafe Opioid Prescribing in Opioid-Naive Patients With Acute Noncancer Pain. *JAMA Health Forum*. Published July 29, 2022. doi:10.1001/jamahealthforum.2022.2263

Data
Data available: Yes
Data types: Deidentified participant data
How to access data: We will make deidentified data available per PCORI policy. A URL for data access is not yet available. Requests may be made to the corresponding author (Kraemer) at kek5@pitt.edu
When available: beginning date: 12-01-2022

Supporting Documents
Document types: None

Additional Information
Who can access the data: To researchers whose proposed use of the data has been approved
Types of analyses: Per PCORI policy, the sharing of data must be in a manner that is appropriate for the nature of the funded research project and that is consistent with applicable privacy, security, and other legal requirements.
Mechanisms of data availability: With a signed data access agreement
Any additional restrictions: Any access will need to comply with PCORI Policy for Data Management and Data Sharing (https://www.pcori.org/sites/default/files/PCORI-Policy-for-Data-Management-and-Data-Sharing.pdf)
