# Supplementary material for: Effect of Different Interventions to Help Primary Care Clinicians Avoid Unsafe Opioid Prescribing in Opioid-Naive Patients With Acute Noncancer Pain: A Cluster Randomized Clinical Trial
Source: JAMA Health Forum. 2022 Jul 29;3(7):e222263. doi: 10.1001/jamahealthforum.2022.2263 (PMC9338412; doi:10.1001/jamahealthforum.2022.2263)
Supplement: Supplement 3. — eTable. Eligible ICD-10 codes for qualifying clinic visit eFigure. Example of Provider Comparison email [file jamahealthforum-e222263-s003.pdf]

Supplemental Online Content

Kraemer KL, Althouse AD, Salay M, et al. Effect of different interventions to help primary care clinicians avoid unsafe opioid prescribing in opioid-naive patients with acute noncancer pain: a cluster randomized clinical trial. *JAMA Health Forum*. 2022;3(7):e222263. doi:10.1001/jamahealthforum.2022.2263

eTable. Eligible ICD-10 codes for qualifying clinic visit
eFigure. Example of Provider Comparison email

This supplemental material has been provided by the authors to give readers additional information about their work.

© 2022 Kraemer KL et al. *JAMA Health Forum.*

eTable. Eligible ICD-10 codes for qualifying clinic visit

o G43.00x, G43.1x, G43.4, G43.8, G44.0x, G44.2x, G44.1 (exclude any of these that use the .x1 or .xx9, indicating intractable headache)<br>o G89.1<br>o M15.xxx, M16.xxx, M17.xxx, M18.xxx, M19.xxx, M22.xxx, M23.xxx, M24.xxx, M25.xxx, M45.xxx, M46.xxx, M47.1xx, M47.2x, M47.81x, M89x, M50.xxx, M51.xxx, M53.xxx, M54.xxx, M65.xxx, M66.xxx, M67.xxx, M70.xxx, M71.xxx, M72.xxx (exclude M72.6), M75.xxx, M76.xxx, M77.xxx, M79.6xx, M94.xxx<br>o R51, R52<br>o S14.xx, S16.xxx, S19.9xx, S23.xxx, S24.xxx, S30.0xx, S30.810, S30.91XA, S40.xxx, S43.xxx, S50.xxx, S53.xxx, S59.9xx, S60.xxx, S63.xxx, S69.9xx, S70.xxx, S73.xxx, S79.9xx, S80.xxx, S83.xxx, S89.8x, S89.9x, S90.xxx, S93.xxx, S99.8. S99.9

© 2022 Kraemer KL et al. *JAMA Health Forum.*

eFigure. Example of Provider Comparison email

Dear Dr. X,

The graph below shows how you compared to peers at other participating clinics on several opioid prescribing metrics. The first metric on the left indicates the proportion of your 15 opioid-naive (defined as no opioid prescriptions in prior 12 months) patients with new non-cancer musculoskeletal pain or headache who received a new opioid prescription in August 2019. The four metrics on the right of the graph are limited to the 16 opioid-naive patients with new non-cancer musculoskeletal pain or headache who you prescribed opioids to since November 2018. For each metric, your data are presented next to the average proportion for providers and the proportion for the lowest 10% of providers at participating clinics. Non-visible bars indicate 0%.

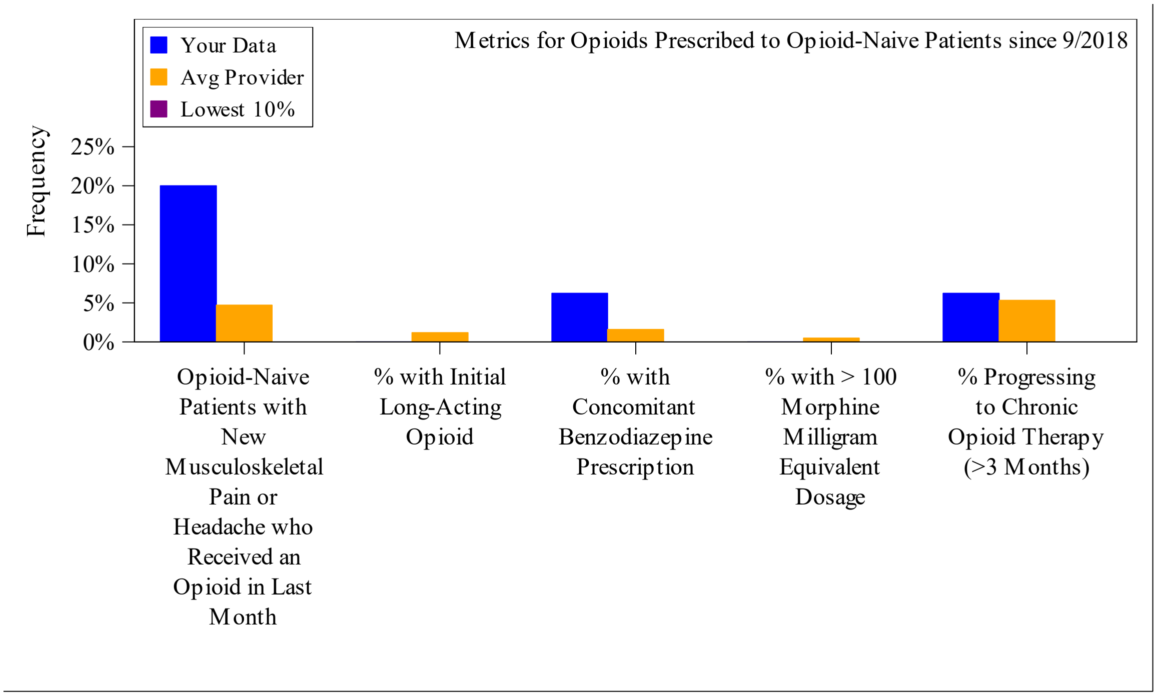

Thank you for your participation,

PCORI Pain Project Team

© 2022 Kraemer KL et al. *JAMA Health Forum.*
